# Genotype turnover and global evolutionary dynamics of G gene duplication variants in human metapneumovirus: insights from prospective surveillance in Nantong, China, 2024–2025

**DOI:** 10.3389/fmicb.2026.1915595

**Published:** 2026-08-12

**Authors:** Xiaolei Ji, Zhenzhen Liu, Xiuli Cai, Ruyue Liang, Lu Chen, Xingpei Ji, Shiyao Chen, Yihua Sun, Chen Guo

**Affiliations:** 1 Nantong Center for Disease Control and Prevention, Nantong, Jiangsu, China; 2 Institute for Applied Research in Public Health, School of Public Health, Nantong University, Nantong, Jiangsu, China; 3 Nantong Chongchuan Center for Disease Control and Prevention, Nantong, Jiangsu, China

**Keywords:** Bayesian phylodynamics, G gene duplication, genotype turnover, human metapneumovirus, O-glycosylation, phylogenetic analysis, whole-genome sequencing

## Abstract

**Background:**

Human metapneumovirus (hMPV) is a leading cause of acute respiratory infections worldwide, yet whole-genome surveillance data remain scarce in eastern China. The evolutionary dynamics of G gene duplication variants and their molecular drivers are poorly characterized.

**Methods:**

From 2024 to 2025, we conducted prospective hMPV surveillance in Nantong, Jiangsu Province, screening 1,602 respiratory specimens by multiplex qPCR. Complete whole-genome sequences were obtained from 26 of 27 positive strains via probe-capture next-generation sequencing, and integrated with 574 global reference genomes. G gene duplication variants (111nt_dup/180nt_dup) were characterized, O-glycosylation sites predicted (NetOGlyc 4.0), and Bayesian phylodynamic analysis (BEAST v1.10.4) performed on the 111nt_dup lineage.

**Results:**

The overall detection rate was 1.7% (27/1,602), with the highest burden in children aged 0–5 years (3.6%) and peak circulation in winter and spring. Phylogenetic analysis revealed a sharp genotype shift among the sequenced strains—from A2.2.2 in 2024 to B2 in 2025 (12/12 vs. 0/14; *p* < 0.001, Fisher’s exact test)—concordant with contemporaneous observations in Shanghai and Beijing. Globally, 111nt_dup prevalence rose from <10% before 2015 to >90% by 2025 (Cochran-Armitage trend test, *p* = 6.86 × 10^−7^), whereas 180nt_dup disappeared after 2021. The 111nt_dup lineage displayed near-complete fixation of serine at G protein position 90 (S90, 98.9% vs. 0% in 180nt_dup; *p* < 0.001). S90 frequency and 111nt_dup prevalence rose in tight synchrony (Pearson’s *r* > 0.95). Bayesian Skyline analysis showed a pandemic-associated population bottleneck (2020–2021) followed by rapid recovery to pre-pandemic levels by late 2022.

**Conclusion:**

The global dominance of 111nt_dup is better explained by lineage-specific molecular selection than neutral drift. Co-occurrence of the 111nt_dup with the predicted S90 O-glycosylation site suggests a model in which predicted glycosylation-driven optimization of the G protein mucin-like domain may enhance fitness, with implications for hMPV vaccine strain surveillance.

## Introduction

Human metapneumovirus (hMPV) is a leading cause of acute respiratory tract infections (ARTIs) across all age groups, with the heaviest disease burden in young children, the elderly, and immunocompromised individuals (Xie et al., 2021; Shafagati and Williams, 2018). Despite its global significance, whole-genome sequence data for hMPV remain scarce, limiting our ability to resolve evolutionary dynamics and genotype turnover (Muñoz-Escalante et al., 2022; Tulloch et al., 2021).

The hMPV attachment glycoprotein (G) is among the most variable hMPV proteins and a central determinant of immune evasion (Nao et al., 2020; Gaunt et al., 2011; Bao et al., 2008). Within the genotype A, the A2.2.2 subclade has two characteristic G gene duplications (111nt_dup and 180nt_dup) which are thought to create clusters of O-glycosylation acceptor sites by extending the mucin-like domain (Groen et al., 2023; Li et al., 2025). Since 2015, independent studies across multiple continents have reported a progressive increase in 111nt_dup prevalence, while 180nt_dup has not been detected in global surveillance data after 2021(Piñana et al., 2014; Pragathi et al., 2024). Whether this asymmetric replacement reflects adaptive molecular evolution or neutral drift has not been resolved.

Previous molecular epidemiological studies of hMPV have relied predominantly on partial G or F gene sequencing, which cannot capture recombination events or resolve fine-scale phylogenetic relationships (Muñoz-Escalante et al., 2022; van den Hoogen et al., 2001; Van Den Hoogen et al., 2004; Wei et al., 2023). In China, most hMPV whole-genome studies have focused on northern cities (Beijing) and central provinces (Henan) (Piñana et al., 2014). From Jiangsu Province—a densely populated region in eastern China—genome-level surveillance data have not been reported prior to the present study (Pragathi et al., 2024).

Here, we report an integrated genomic epidemiological investigation of hMPV based on two-year prospective surveillance in Nantong, Jiangsu Province (2024–2025). From 1,602 clinical respiratory specimens, we identified 27 hMPV-positive cases and obtained 26 complete whole-genome sequences. We reconstructed a global phylogenetic dataset of 600 full-length hMPV genomes, characterized G gene duplication variants within A2.2.2, analyzed O-glycosylation patterns, and performed Bayesian phylodynamic analysis of the 111nt_dup lineage. Our results reveal a marked genotype turnover (A2.2.2 to B2) across consecutive seasons among the sequenced strains and suggest that the rise of 111nt_dup to global dominance is consistent with lineage-specific molecular selection—with a co-occurring predicted O-glycosylation site (S90) as a candidate molecular driver.

## Materials and methods

### Study population and specimen collection

This study was conducted between 2024 and 2025, and a total of 1,602 clinical respiratory specimens were enrolled. The study subjects included patients with influenza-like illness (ILI) and severe acute respiratory infection (SARI). The specimen types consisted of nasopharyngeal swabs or bronchoalveolar lavage fluid (BALF). After collection, all specimens were placed in viral transport medium (VTM) and stored at −80 °C until further nucleic acid extraction.

### Nucleic acid extraction

Nucleic acid extraction was performed in the Central Microbiology Laboratory. Briefly, 300 μL of the sample supernatant was aspirated, and total nucleic acids (including RNA and DNA) were extracted using an automatic nucleic acid extractor (Liferiver, Shanghai, China, Model: EX3600Plus). The extraction process was carried out with the matching nucleic acid extraction kit (Liferiver, Shanghai, China, Cat. No.: Z-M-0092-96). All experimental operations were strictly performed in accordance with the manufacturer’s standard operating procedures (SOPs). The extracted nucleic acid products were immediately used for subsequent downstream detection without delay.

### hMPV screening by real-time quantitative PCR

Screening for human metapneumovirus (hMPV) was conducted using a 17-plex respiratory pathogen nucleic acid detection kit (BioGerm, Shanghai, China, Cat. No.: SJ-HX-866-2). The preparation of the reaction system, setting of amplification parameters, and interpretation of detection results were all performed in strict accordance with the manufacturer’s kit instructions. Amplification reactions were carried out on a 7,500 real-time quantitative PCR (qPCR) instrument (Applied Biosystems, ABI, Foster City, CA, United States).

### Whole genome sequencing and library construction

Whole genome sequencing was performed on the 27 hMPV-positive samples identified via the above screening. Library construction was carried out using the iGeneTech^®^ RNA Pathogen Microbial Library Construction and Capture Kit (iGeneTech, Beijing, China, Cat. No.: PROT241101), with all experimental procedures strictly following the manufacturer’s instructions. Briefly, the extracted total RNA was first subjected to fragmentation, followed by reverse transcription and second-strand cDNA synthesis. Pre-PCR amplification was performed after the completion of end repair and adapter ligation.

To improve sequencing efficiency and reduce background noise, liquid-phase probe hybridization capture technology was applied to enrich the target hMPV genomic sequences. TargetSeq^®^ specific probes (iGeneTech, Beijing, China) were used for the hybridization capture process. The probe panel was designed against conserved and variable regions spanning all known hMPV subtypes (A1, A2.1, A2.2.1, A2.2.2, B1, B2), providing uniform capture coverage across genotypes A and B, which precludes systematic genotype-specific binding bias in the recovery rates reported here. The captured libraries were subjected to Post-PCR amplification, purification, and quality control (QC). The final qualified libraries were sequenced with paired-end 150 bp (PE150) mode on the Illumina MiSeq sequencing platform (Illumina, San Diego, CA, United States).

### Bioinformatics analysis

Raw sequencing data were first evaluated for quality using FastQC. Adapter sequences were trimmed and low-quality reads were filtered with fastp (v0.12.1) (Chen et al., 2018). The filtered reads were then aligned to the human reference genome (GRCh38) via Bowtie2 (v2.5.4) (Langdon, 2015) to eliminate host contamination. The resulting high-quality non-host reads were assembled *de novo* using MEGAHIT (v1.2.9) (Li et al., 2015). Contigs from the assembly were searched against the NCBI pathogen database with blastn. The most matching hMPV sequence, selected based on bit-score and coverage, served as the reference sequence. Subsequently, high-quality reads were realigned to this reference using BWA (v0.7.18) (Li and Durbin, 2009). The alignments were sorted and deduplicated with SAMtools (v1.20) (Li et al., 2009). Consensus sequences were generated using iVar (v1.4.2) (Grubaugh et al., 2019), with minimum thresholds applied for sequencing depth (10×) and base quality. Sequences achieving less than 90% ≥ 10 × coverage of the target genome region were excluded from subsequent molecular evolutionary analyses. All assembled whole-genome sequences were manually curated.

### Phylogenetic analysis and genotyping

**Figure 1:**
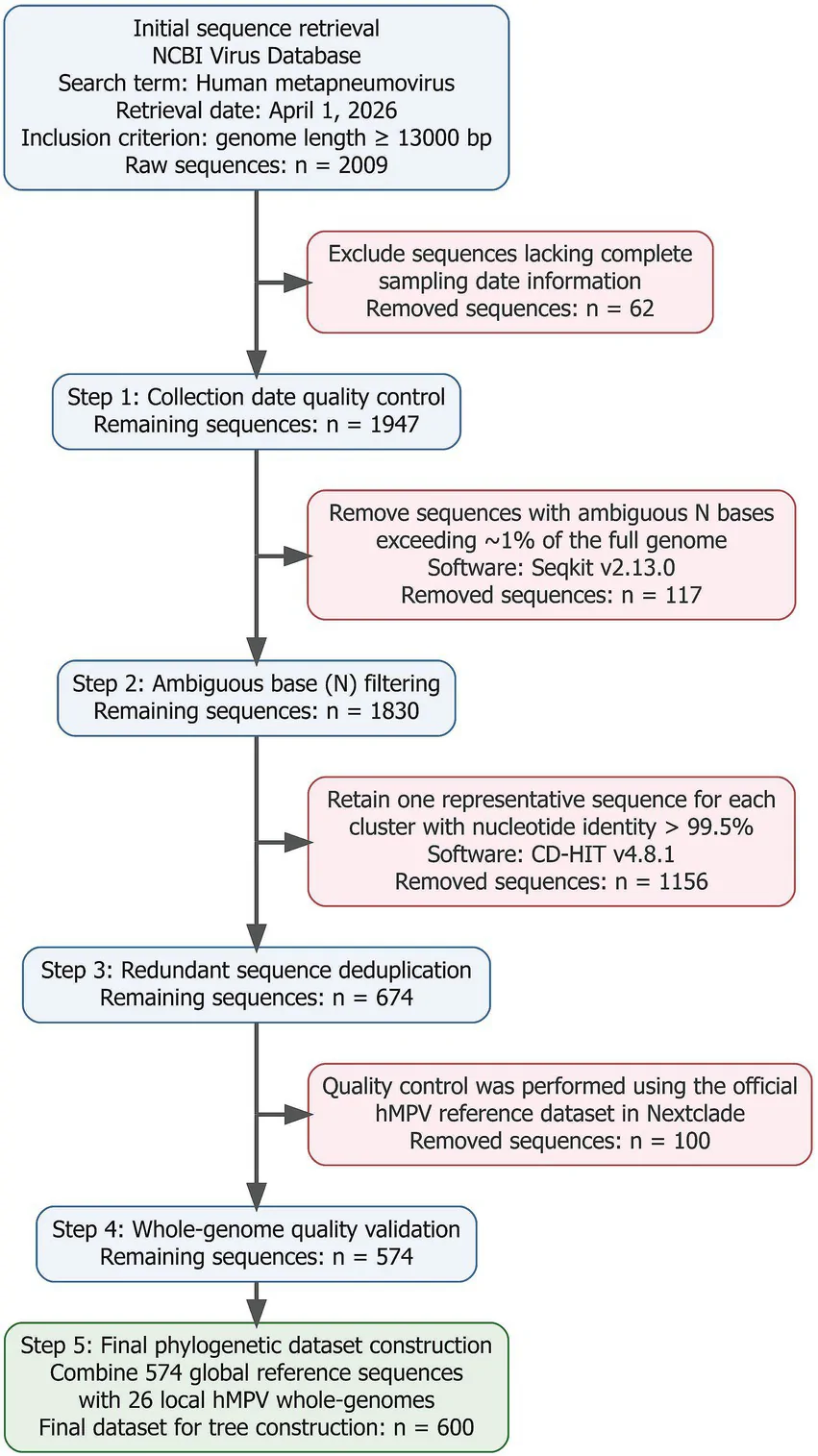
Workflow for the construction of the hMPV full-genome phylogenetic analysis dataset.

To elucidate the genetic classification and evolutionary relationship of the 26 newly assembled complete hMPV genomes in this study, we first constructed a high-quality full-genome dataset for phylogenetic reconstruction. Full-length hMPV genome sequences (≥13,000 bp, >97% genome completeness) were retrieved from the NCBI Virus database on April 1, 2026, and screened through a multi-step quality control and dereplication pipeline (Figure 1). Sequences lacking complete sampling dates or containing >1% ambiguous (N) bases were excluded. Near-identical sequences (>99.5% nucleotide identity) were dereplicated using CD-HIT v4.8.1, retaining one representative per cluster. Remaining sequences were verified for whole-genome quality using Nextclade with the official hMPV reference dataset. This workflow produced 574 qualified global reference sequences. Together with the 26 local complete hMPV genomes assembled in this study, these formed the final phylogenetic analysis dataset of 600 full-length genomes.

Multiple sequence alignment of the full-length genome sequences was performed using MAFFT (v7.525) (Katoh and Standley, 2013) with the “--auto” strategy. The aligned sequences were then trimmed using the “-automated1” (auto) automated mode in TrimAI (v1.5.1) (Capella-Gutiérrez et al., 2009), to remove poorly aligned sites, high-gap regions and phylogenetically uninformative redundant fragments. No sequences were excluded during this process, and the total number of sequences remained unchanged for subsequent tree construction. Maximum Likelihood (ML) phylogenetic trees were reconstructed using IQ-TREE 2 (v2.3.6) (Minh et al., 2020). For each phylogenetic reconstruction (including the full-genome main tree and subtype-specific subtrees), the optimal nucleotide substitution model was automatically selected by ModelFinder based on the Bayesian Information Criterion (BIC). Branch support was evaluated using the ultrafast bootstrap method with 1,000 replicates. The final phylogenetic trees were visualized, annotated and graphically refined using the online bioinformatics platform TVBOT (Xie et al., 2023).^1^ Genotype assignment of local strains was determined based on tree topology, established standard hMPV genotyping nomenclature, and a reliable clade classification threshold of >70% bootstrap support.

The workflow includes four core modules of quality control, filtering and dereplication, starting from raw sequences retrieved from the NCBI Virus database, to obtain high-quality global representative hMPV reference sequences, which were finally integrated with the local genomes generated in this study to form the final dataset for phylogenetic analysis.

### G gene duplication variant analysis

For the characterization of G gene duplication variants, full-length G gene coding sequences were extracted from the quality-controlled A2.2.2 subtype global reference sequences and locally generated sequences described above. Sequences were classified into three groups: 111nt_dup, 180nt_dup, and non-dup, based on the presence of the target duplication events in the G gene. Multiple sequence alignment of the duplication region and its flanking sequences was performed using MAFFT v7.525, and nucleotide conservation across the aligned region was visualized using WebLogo 3, with bit values ranging from 0 to 2 to quantify the degree of base conservation at each position. The fixed insertion site of 111nt_dup and 180nt_dup, both of which are nucleotide duplication events, was annotated based on the consensus sequence of non-duplicated A2.2.2 strains. For temporal prevalence analysis, all included A2.2.2 sequences were stratified by the year of sample collection. The annual prevalence of 111nt_dup and 180nt_dup was calculated as the percentage of variant-positive sequences relative to the total number of A2.2.2 sequences in the corresponding year. The temporal trend in 111nt_dup prevalence was assessed using a two-sided Cochran-Armitage trend test, with a *p* < 0.05 considered statistically significant.

### G protein O-glycosylation site prediction and evolutionary analysis

Potential O-glycosylation sites of the full-length G protein were predicted using the NetOGlyc 4.0^2^ (Steentoft et al., 2013) server with a default threshold of 0.5. Only sites located within the extracellular ectodomain (amino acids 54 to the C-terminus) (Leyrat et al., 2014) were included in subsequent analyses, as the intracellular and transmembrane domains do not undergo glycosylation. Multiple sequence alignment of the G protein amino acid sequences was performed using MAFFT v7.525 with the --auto strategy. A total of 219 high-quality full-length G protein sequences were included in the final alignment, after excluding 24 sequences with frameshift mutations or ambiguous gaps that disrupted correct amino acid translation. The alignment was manually inspected and corrected to ensure accurate homology matching. The 111nt_dup and 180nt_dup insertion zones were defined based on the characteristic gap patterns observed in non-duplicated strains within the corrected alignment. For group-level conservation analysis, the glycosylation frequency at each alignment position was calculated as the percentage of strains within a variant group that contained a predicted O-glycosylation site at that position. For the co-evolutionary analysis of the key glycosylation site at alignment position 90, the annual frequency of serine at this position was calculated and compared with the annual prevalence of 111nt_dup and 180nt_dup variants derived from the global prevalence dataset. Statistical differences in glycosylation frequency at position 90 between variant groups were assessed using Fisher’s exact test, with *p* < 0.001 considered statistically significant.

### Bayesian phylodynamic analysis

Bayesian phylodynamic analysis was performed on the full-length G gene nucleotide sequences of the 111nt_dup strains (*n* = 93). The evolutionary rate and historical effective population size (Neτ) were estimated using BEAST v1.10.4 (Baele et al., 2025). The nucleotide substitution model was set to GTR + G + I. An Uncorrelated Lognormal Relaxed Clock model was employed. The Coalescent: Bayesian Skyline tree prior was selected to reconstruct past population dynamics. The Markov Chain Monte Carlo (MCMC) algorithm was run for 100,000,000 generations, with sampling every 10,000 generations. Convergence of the MCMC chains was assessed using Tracer v1.6 (Rambaut et al., 2018), ensuring that the Effective Sample Size (ESS) for all parameters exceeded 200 after a 10% burn-in. The Bayesian Skyline Plot was rendered using the ggplot2 package in R software (v4.6.0).

## Results

### Epidemiological characteristics

A total of 1,602 respiratory clinical samples were included in this study (Table 1). Following screening by fluorescent PCR, 27 hMPV-positive cases were detected, yielding an overall positive rate of 1.7%. The positive rate was 1.2% (11/913) in males and 2.3% (16/689) in females. Statistical analysis revealed no significant difference in the hMPV detection rate between different genders (χ^2^ = 2.959, *p* = 0.085).

**Table 1:** Epidemiological characteristics of the enrolled hMPV-positive patients in Nantong from 2024 to 2025.

Characteristic	Frequency of enrolled patients	Frequency of HMPV-positive patients	Positive rate of HMPV (%)	χ^2^ value	*p*-value
Sex				2.959	0.085
Male	913	11	1.2%		
Female	689	16	2.3%		
Age				15.109	0.002
0 ~ 5	439	16	3.6%		
6 ~ 14	374	5	1.3%		
15 ~ 64	310	1	0.3%		
>65	479	5	1.0%		
Case type				6.728	0.009
ILI	978	23	2.4%		
SARI	624	4	0.6%		
Year				0.089	0.765
2024	817	13	1.6%		
2025	785	14	1.8%		
Season				32.561	<0.001
Spring	246	11	4.5%		
Summer	762	2	0.3%		
Autumn	200	0	0.0%		
Winter	394	14	3.6%		

A statistically significant difference in detection rates was observed among different age groups (χ^2^ = 15.109, *p* = 0.002). The highest positive rate was 3.6% (16/439) in the 0–5 years preschool children group. Pairwise comparisons with Bonferroni correction showed that the detection rate in the preschool children group was significantly higher than that in all other groups. Specifically, significant differences existed between the preschool children group and the 6–14 years children group (*p* = 0.039), the 15–64 years young and middle-aged adults group (*p* = 0.003), and the >65 years elderly group (*p* = 0.008). The positive rates for the 6–14 years, 15–64 years, and >65 years groups were 1.3, 0.3, and 1.0%, respectively. No statistically significant differences were found among these three groups (*p* > 0.05).

The distribution across case types showed a significant difference (χ^2^ = 6.728, *p* = 0.009). The positive rate among ILI cases was 2.4% (23/978), significantly higher than the 0.6% (4/624) among SARI cases. Regarding temporal distribution, the positive rates for 2024 and 2025 were 1.6 and 1.8%, respectively. No significant fluctuation in detection rates was observed between the years (χ^2^ = 0.089, *p* = 0.765).

HMPV circulation exhibited a strong seasonal pattern (χ^2^ = 32.561, *p* < 0.001). Spring and winter were the primary epidemic peaks. The positive rate was 4.5% (11/246) in spring and 3.6% (14/394) in winter. Only two positive cases (0.3%) were detected in summer, and no positive cases were found in autumn. Results from pairwise comparisons indicated that the detection rates in both winter and spring were significantly higher than those in summer and autumn (*p* < 0.05). No statistically significant difference was found between winter and spring (*p* = 0.560), nor between summer and autumn (*p* = 0.468).

### Whole-genome sequencing and assembly results

High-throughput sequencing was performed on 27 hMPV-positive samples identified through screening. Sequencing was conducted on the Illumina MiSeq platform using a probe capture-based library construction strategy. The Clean Data (effective data after adapter and low-quality read removal) per sample ranged from 0.51 Gb to 2.66 Gb, with an average of ~1.21 Gb, providing a sufficient data foundation for subsequent genome assembly.

An assembly strategy combining *de novo* assembly with reference sequence mapping was employed. Through this pipeline, high-quality whole-genome sequences were successfully obtained for 26 hMPV strains. The assembled genome length of these strains ranged from 13,165 to 13,398 bp, with an average of ~13,280 bp. For the 26 high-quality strains (excluding HMPV-9), the average sequencing depth exceeded 70,000 × (minimum: 17,344×), and the average ≥10 × coverage of the target region reached over 99.8%, confirming high sequencing quality and completeness.

Although a near-full-length scaffold (13,403 bp) was generated for HMPV-9, this sample showed significantly low ≥10 × coverage (72.21%) due to a low proportion of target reads. This was likely associated with low original viral load or nucleic acid degradation in the sample. Consequently, HMPV-9 was excluded from subsequent molecular evolutionary analyses, which focused solely on the remaining 26 high-quality genomes. The assembled whole-genome sequences of the 26 high-quality strains have been submitted to GenBank (Table 2).

**Table 2:** Statistics of sequencing data and assembly quality for HMPV samples.

Sample ID	Clean data (Gb)	Mean depth (×)	≥10 × coverage (%)	Assembled length (bp)	Accession no.
HMPV-1	0.87	61542.43	99.69	13,395	PZ529211
HMPV-2	0.74	45046.25	99.65	13,281	PZ529212
HMPV-3	0.88	58681.55	99.94	13,395	PZ529213
HMPV-4	0.96	62737.45	99.68	13,397	PZ529214
HMPV-5	0.85	55034.98	99.63	13,387	PZ529215
HMPV-6	0.60	17344.14	99.72	13,349	PZ529216
HMPV-7	1.07	62282.05	99.83	13,264	PZ529217
HMPV-8	1.39	95344.07	99.78	13,265	PZ529218
HMPV-9	0.55	720.22	72.21	13,403	/
HMPV-10	2.66	184792.34	99.90	13,238	PZ529219
HMPV-11	0.64	45377.66	99.80	13,270	PZ529220
HMPV-12	0.51	38653.05	99.66	13,398	PZ529221
HMPV-13	2.00	137356.26	99.91	13,278	PZ529222
HMPV-14	1.50	100509.46	100.00	13,280	PZ529223
HMPV-15	1.83	126551.65	99.90	13,240	PZ529224
HMPV-16	1.59	99012.15	100.00	13,251	PZ529225
HMPV-17	1.36	71945.82	100.00	13,251	PZ529226
HMPV-18	1.16	72543.39	100.00	13,250	PZ529227
HMPV-19	1.46	66876.58	100.00	13,251	PZ529228
HMPV-20	1.71	88021.75	100.00	13,246	PZ529229
HMPV-21	2.01	93705.64	100.00	13,253	PZ529230
HMPV-22	1.33	66876.32	99.86	13,250	PZ529231
HMPV-23	1.30	81112.85	99.99	13,252	PZ529232
HMPV-24	1.21	76213.45	99.97	13,165	PZ529233
HMPV-25	0.98	62324.53	100.00	13,250	PZ529234
HMPV-26	0.99	64012.83	100.00	13,248	PZ529235
HMPV-27	1.08	64125.78	100.00	13,263	PZ529236

1. Clean Data: Refers to the high-quality sequencing data retained after trimming adapter sequences and filtering low-quality reads; 2. Mean Depth: Denotes the average sequencing depth across the target genome.; 3. ≥ 10 × Coverage: Represents the percentage of genomic positions within the target region that achieve a sequencing depth of at least 10-fold.; 4. Assembled Length: Refers to the total length of the final consensus sequence obtained through the assembly pipeline.

### Phylogenetic analysis and genotyping

**Figure 2:**
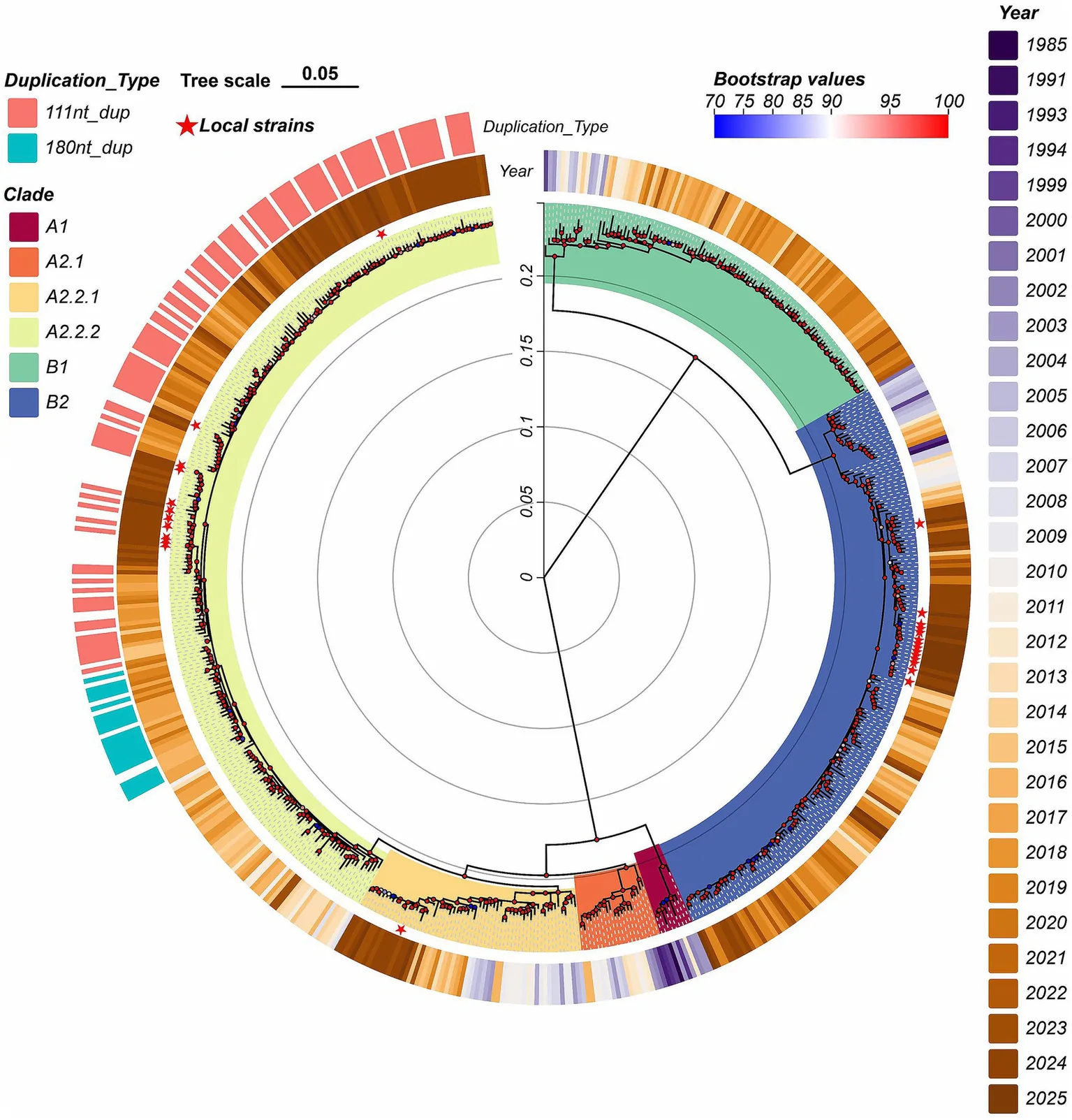
Maximum likelihood phylogenetic tree based on 600 full-length hMPV genomes.

Maximum likelihood (ML) phylogenetic trees were constructed based on the final dataset of 600 full-length hMPV genomes (detailed in Figure 2). Phylogenetic topology showed that all sequences were clearly divided into 6 major clades consistent with the internationally standard hMPV genotyping nomenclature, namely subtypes A1, A2.1, A2.2.1, A2.2.2, B1 and B2. Bootstrap support values for all major genotype clades exceeded 90%, confirming the stability of the phylogenetic topology and reliability of the genotyping results.

Genotyping of the 26 high-quality full-length hMPV genomes generated via direct sequencing of clinical samples in this study yielded clear and consistent results: all 12 sequences detected in 2024 belonged to genotype A, with 1 assigned to subtype A2.2.1 and 11 to subtype A2.2.2; all 14 sequences detected in 2025 were classified into genotype B, subtype B2. A striking annual genotype shift was observed in locally circulating hMPV strains: genotype A dominated in 2024, and no genotype A strain was detected among the 14 sequenced cases from 2025, all of which belonged to subtype B2.

Annotation of the phylogenetic tree revealed that the 111nt_dup and 180nt_dup variants in the G gene were exclusively distributed in the A2.2.2 subclade of genotype A, with no such duplications detected in any other subtypes. Of the 11 A2.2.2 sequences detected in 2024, 7 carried the 111nt_dup, corresponding to a positive rate of 63.6% within this subtype. The remaining 4 A2.2.2 sequences and 1 A2.2.1 sequence had no detectable duplications. No nucleotide duplications in the G gene were found in any of the 14 B2 sequences from 2025.

Subtype-specific phylogenetic trees for the A2.2.1, A2.2.2 and B2 clades are provided in Supplementary Figures S1–S3, which confirmed that all local hMPV sequences were genetically closely related to global epidemic strains, with no independent endemic transmission cluster identified in Nantong.

The phylogenetic tree was reconstructed using IQ-TREE 2, with the optimal nucleotide substitution model selected by ModelFinder based on the Bayesian Information Criterion (BIC). Branch reliability was evaluated by 1,000 ultrafast bootstrap replicates, and bootstrap support values >70% were labeled on the major clades. A total of 600 full-length hMPV genomes were included in this analysis, comprising 574 global reference sequences and 26 high-quality sequences generated in this study via direct clinical sample sequencing. The 26 local sequences from this study were highlighted with red solid stars. Sequences carrying the 111 and 180 nt dup in the G gene were marked with pink and blue solid squares, respectively. The six major subtypes (A1, A2.1, A2.2.1, A2.2.2, B1, B2) were annotated with colored blocks on the left side of the tree.

### Characteristics and global prevalence trends of G gene duplication variants

**Figure 3:**
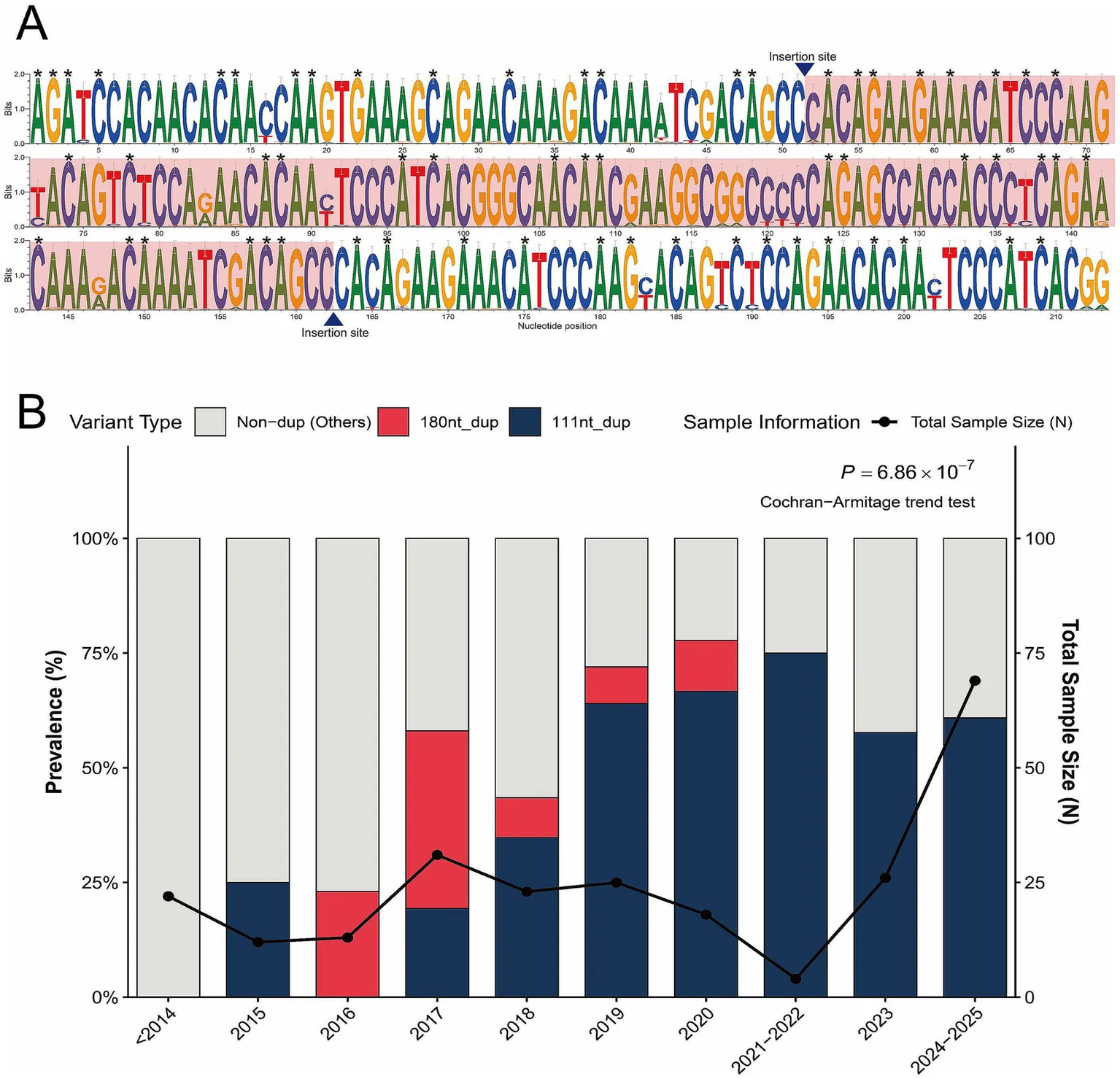
G-gene insertion locus conservation and global temporal prevalence of hMPV A2.2.2 duplication variants. **(A)** WebLogo visualization of nucleotide conservation within 51 bases upstream and downstream of the fixed 111nt_dup insertion site. The Y-axis bit values (0–2) reflect the conservation degree at each locus, where asterisks denote 100% conserved positions and a blue triangle marks the duplication insertion breakpoint. **(B)** Stacked bar chart tracking the global annual prevalence profiles of 111nt_dup, 180nt_dup, and non-duplicated hMPV A2.2.2 lineages from 2008 to 2025. The left Y-axis denotes the relative prevalence of each variant (%), the right Y-axis shows the total number of A2.2.2 full-length genome sequences included per year, with corresponding values represented by a black solid line with circular markers. The *p*-value for the temporal increasing trend of 111nt_dup prevalence was derived from a two-sided Cochran-Armitage trend test.

Sequence conservation analysis of the G gene region flanking the duplication insertion site showed that the flanking sequences were highly conserved across all included A2.2.2 strains, with multiple nucleotide positions exhibiting 100% conservation in the global dataset (Figure 3A). This result confirmed that 111nt_dup arose from a fixed, locus-specific insertion event, rather than random genomic rearrangements. The temporal prevalence analysis graphed in Figure 3B revealed that the global A2.2.2 strains that were duplicated underwent a shift in dominant variant during the time period. The 180nt_dup variant was only temporarily established between 2016 and 2020, showing an annual maximum prevalence of 38.71% in 2017 and not detected in global A2.2.2 strains after 2021. By contrast, 111nt_dup first identified in 2015, using the global A2.2.2 strains was established may over time (Cochran-Armitage trend test, χ^2^ = 24.65, df = 1, *p* = 6.86 × 10^−7^). After 2019, the frequency of 111nt_dup remained stabilized above 50%. Subsequently, 111nt_dup gradually displaced the other variants and became the predominant genotype in global A2.2.2. The early evolutionary features of subtype A2.2.2 are related to the lack of duplication of variants in the strains collected before 2014.

#### O-Glycosylation landscape of hMPV A2.2.2 subtype and co-evolution of the Pos 90 glycosylation anchor

**Figure 4:**
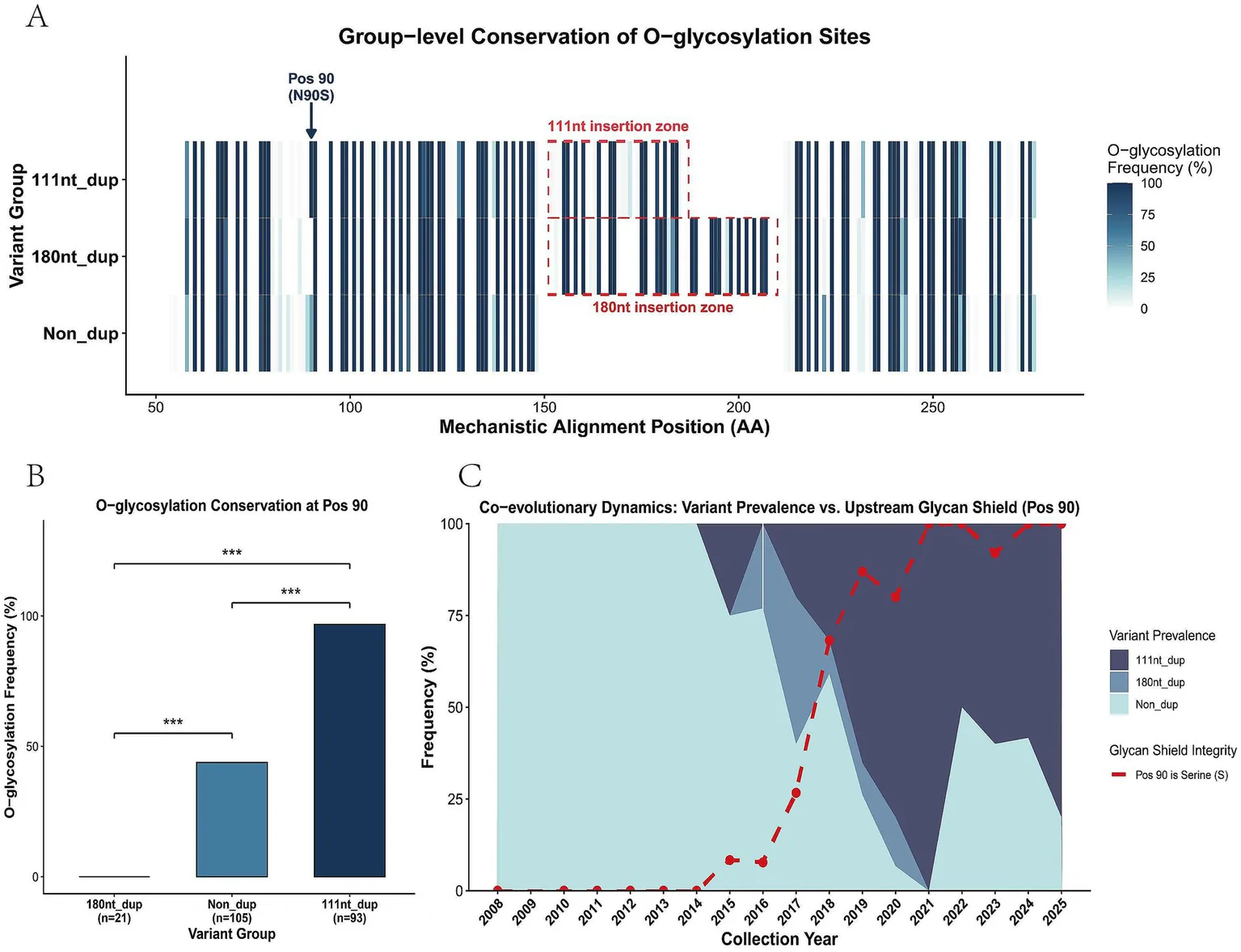
O-glycosylation landscape of hMPV A2.2.2 subtype strains and co-evolutionary dynamics of the Pos 90 glycosylation anchor. **(A)** Heatmap showing the group-level conservation of predicted O-glycosylation sites across the G protein ectodomain. Rows represent the three variant groups (111nt_dup, 180nt_dup, non-dup), and columns represent the mechanistic alignment position of amino acids. Color intensity indicates the percentage of strains within each group containing a predicted O-glycosylation site at the corresponding position. The 111 nt and 180 nt insertion zones are marked with shaded boxes, and the key polymorphic anchor site (Pos 90) is indicated by an arrow. **(B)** Bar chart showing the frequency of the O-glycosylation site at Pos 90 in the three variant groups. ***p < 0.001, two-sided Fisher’s exact test. **(C)** Line chart showing the temporal correlation between the annual frequency of the S90 allele and the annual prevalence of 111nt_dup and 180nt_dup variants from 2008 to 2025.

Comparative mapping of predicted O-glycosylation sites across the G protein revealed a clear lineage-specific structural partition within the hMPV A2.2.2 subclade (Figure 4A). This divergence centered on the C-terminal insertion segments and their immediate upstream flanking regions. The 111nt_dup region hosted a dense, highly conserved cluster of 13 O-glycosylation sites shared across all 93 expansion strains. Consistent with its greater sequence length, the 180nt_dup insertion pocket contained 24 high-frequency O-glycosylation sites, whereas no O-glycosylation was detected at these positions among the 105 non-duplicated genomes. Alignment position 90 (Pos 90), situated directly upstream of the insertion threshold, showed divergent predicted O-glycosylation patterns among the three variant cohorts.

Analysis of all 219 full-length G sequences showed that the predicted glycosylation status at Pos 90 correlates with a near-dichotomous allelic distribution (Figure 4B). According to NetOGlyc 4.0 predictions, a serine (S90) residue exceeded the prediction threshold for O-glycosylation, whereas an asparagine (N90) fell below threshold. The non-duplicated lineage retained an intermediate ancestral polymorphism at this locus, with a baseline predicted O-glycosylation frequency of 43.8%. Both duplication lineages lost this polymorphism, but in opposite directions: the 180nt_dup variants were fixed for the N90 allele (0% predicted glycosylation), whereas the 111nt_dup lineage showed near-complete fixation of the S90 substitution, with a predicted glycosylation frequency of 98.9%. Two-sided Fisher’s exact tests indicated that these differences in amino acid distribution were significant (non-dup vs. 180nt_dup, 180nt_dup vs. 111nt_dup, and non-dup vs. 111nt_dup; all *p* < 0.001).

Tracking these molecular signatures longitudinally from 2008 to 2025 revealed a tight temporal coupling between S90 allele frequencies and the expanding ecological footprint of the 111nt_dup variant (Figure 4C). Before 2015, the S90 allele remained below 10% globally, reflecting the fluctuating polymorphic baseline of historical non-duplicated strains. After the initial detection of 111nt_dup in 2015, the S90 allele rose sharply in parallel with the variant’s prevalence, reaching 98.5% by 2025. In contrast, the N90-restricted 180nt_dup lineage steadily contracted and disappeared from global sequence data after 2021.

### Bayesian phylodynamic analysis and demographic history of the 111nt_dup lineage

Bayesian phylodynamic analysis of the G gene from the 111nt_dup lineage estimated an overall evolutionary rate of 4.438 × 10^−3^ substitutions/site/year (95% HPD: 3.445 × 10^−3^–5.483 × 10^−3^). The Time to the Most Recent Common Ancestor (tMRCA) of the 111nt_dup lineage was dated to 2013.43 (95% HPD: 2012.15–2014.52).

**Figure 5:**
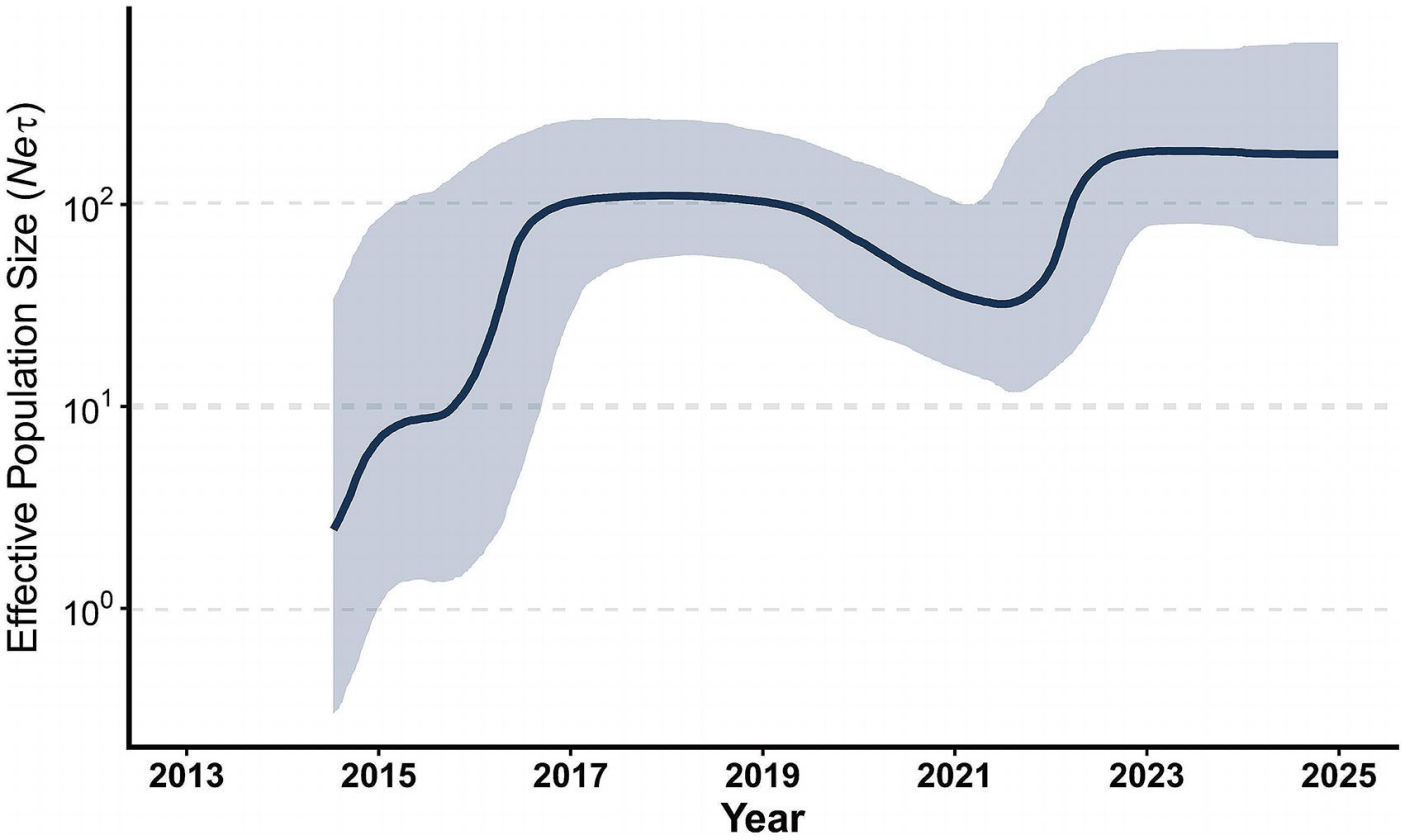
Evolutionary demographic history of the globally dominant hMPV A2.2.2 111nt_dup lineage.

The demographic history of this lineage was reconstructed using a Bayesian Skyline Plot (BSP) (Figure 5). The estimated trajectory of the effective population size (Neτ) showed a fluctuating trend that can be characterized by six sequential demographic intervals from 2014 to 2025.

From its root origin in mid-2014, the lineage remained at a low baseline population size through early 2015 (Phase I). Beginning in 2015, the Neτ expanded rapidly and steeply, ending by late 2016 (Phase II). After this initial expansion, the Neτ remained at a high, stable plateau from 2017 through 2019 (Phase III). This equilibrium was disrupted by a sharp population bottleneck, with a steep decline in Neτ from early 2020 through mid-2021 (Phase IV). After this contraction, a second wave of rapid exponential expansion followed from late 2021 through mid-2022 (Phase V). From late 2022 to 2025, growth halted, and the lineage entered a second equilibrium at a level comparable to its pre-bottleneck peak (Phase VI).

The Bayesian Skyline Plot (BSP) shows the temporal dynamics of the effective population size (Neτ) from the lineage’s root origin in 2013 to 2025. The solid black line marks the median Neτ estimate on a logarithmic scale, and the shaded area covers the 95% highest posterior density (HPD) interval. The reconstructed trajectory traces six demographic phases. After an initial low-level emergence from mid-2013 to early 2015 (Phase I), the population underwent a rapid exponential expansion through late 2016 (Phase II) and then settled into a high, stable plateau lasting from 2017 to 2019 (Phase III). A sharp bottleneck followed, with a steep decline in Neτ from early 2020 to mid-2021 (Phase IV). A second rapid expansion then occurred from late 2021 to mid-2022 (Phase V), after which the lineage reached a second equilibrium at levels comparable to the pre-bottleneck peak (Phase VI, late 2022 to 2025).

## Discussion

In this study, we analyzed the epidemiological features and evolutionary dynamics of human metapneumovirus (hMPV) using two-year prospective surveillance in Nantong, eastern China (2024–2025). The overall local detection rate was 1.7%, lower than rates from hospital-based surveillance in Beijing (7.9%) (Cong et al., 2022), Nanjing (4.7%) (Min et al., 2024), and Qingdao (3.8%) (Liu et al., 2018). These differences likely reflect both regional variation in circulation intensity and methodological heterogeneity—including enrollment criteria, detection assays, and sampling frames—across studies. The Nantong surveillance framework captured representative local hMPV circulation across seasons and patient groups, providing an adequate basis for the molecular analyses below.

Within this cohort, hMPV conformed to its known profile as a predominantly pediatric pathogen (Lamichhane et al., 2023): preschool children aged 0–5 years bore the highest infection burden (3.6%), while the detection rate declined sharply in older age groups. Positivity was significantly higher among outpatients with influenza-like illness (ILI) than among patients with severe acute respiratory infection (SARI). This difference partly reflects the larger number of ILI presentations, but it also suggests that hMPV in this setting primarily caused mild-to-moderate upper respiratory tract infection rather than severe pneumonia, consistent with findings from a comparable German study (Borgmann et al., 2026). The pronounced winter–spring seasonality we observed matches the classic circulation pattern of pneumoviruses in temperate regions (García-Arroyo et al., 2022; Berry et al., 2024). Despite these well-defined seasonal and clinical features, however, the factors driving the temporal dynamics and lineage composition of hMPV circulation in this region are still unclear. To address this gap, we performed whole-genome sequencing and evolutionary analyses of the locally circulating strains.

The phylogenetic reconstruction revealed a marked genotype turnover across the two consecutive epidemic seasons among the sequenced strains. Genotype A, predominantly A2.2.2, accounted for all 12 sequenced strains in 2024, yet no genotype A strain was detected among the 14 sequenced cases from 2025, all of which belonged to B2. This binary shift is not an isolated local event. Contemporaneous studies from Shanghai and Beijing, both published in 2025, describe an essentially identical transition: in Shanghai, a genotype shift from the A2.2.2 111 nt-dup variant to B2 occurred in December 2024, triggering an outbreak that lasted through April 2025 (Zhu et al., 2026); in Beijing, B2 re-emerged with marked predominance (98.3%) in the 2024–2025 season, displacing the previously circulating A2.2.2 111 nt-dup lineage (Li et al., 2025). The close temporal concordance across Nantong, Shanghai, and Beijing indicates a coordinated genotype turnover affecting much of eastern and northern China within the same season. This phenomenon has historical precedent. A decade-long surveillance study in Henan province documented an alternating “AAABBA” pattern between 2017 and 2022, in which genotype A dominated during the first three seasons, genotype B during the next two, and genotype A returned in the final season (Xie et al., 2024). Internationally, periodic broad genotype turnover has been reported in Kenya and Japan (Owor et al., 2016; Shirato et al., 2024), indicating that alternating dominance is a fundamental feature of hMPV population dynamics, not a regional peculiarity. The binary nature of the shift we observed (A-only in one season, B-only in the next) is somewhat sharper than the co-circulation pattern typical of multi-year surveillance, and may partly reflect the modest number of local sequences, which could undersample minority genotypes at low frequency. The temporal boundary between the two genotype regimes, however, was clearly defined in our dataset and is corroborated by independent observations elsewhere in China.

The 111nt_dup and 180nt_dup variants in the G gene represent a second feature central to the molecular evolution of hMPV. Consistent with the global hMPV dataset, both variants were detected exclusively within the A2.2.2 subclade in our collection (Nao et al., 2020; Groen et al., 2023). Among the 11 local A2.2.2 strains from 2024, the 111nt_dup variant accounted for 63.6% (7/11), while the 180nt_dup was not detected among the 11 local A2.2.2 strains. The cyclical genotype shift to B2 in 2025 temporarily displaced genotype A from local circulation, a pattern characteristic of hMPV inter-genotypic dynamics. More notable is the asymmetric distribution within A2.2.2 itself. The predominance of the 111nt_dup and the concurrent disappearance of the 180nt_dup from the local A2.2.2 population reflect a broader trend: global surveillance has documented a steady rise of the 111nt_dup in A2.2.2 strains across Asia, Europe, and South America since 2016, with this variant progressively replacing both the wild-type A2.2.2 and the 180nt_dup counterpart in multiple regions (Muñoz-Escalante et al., 2022; Saikusa et al., 2014). The consistency between local and global patterns suggests the competitive advantage of the 111nt_dup is a reproducible evolutionary outcome rather than a regional phenomenon. The structural and functional basis of this advantage, including the amino acid substitutions and O-glycosylation modifications associated with the duplication, merits closer study.

The global prevalence trajectories of the two G gene duplication variants place our local observations in a broader context and highlight a clear evolutionary asymmetry. In the global A2.2.2 dataset, the 180nt_dup variant followed a transient epidemiological course: it appeared, peaked at 38.71% in 2017, and then declined steadily until it was no longer detected after 2021 (Groen et al., 2023). By contrast, the 111nt_dup, first identified in 2015 (Piñana et al., 2014), increased progressively and significantly in prevalence over time (Cochran-Armitage test, *p* = 6.86 × 10^−7^), stabilized above 50% after 2019, and eventually displaced other variants to become the dominant form within the A2.2.2 subtype (Muñoz-Escalante et al., 2022). The strict conservation of the flanking sequences surrounding the insertion site across all A2.2.2 strains examined further indicates that the 111nt_dup arose from a single, fixed mutational event rather than from recurrent genomic rearrangement.

The divergent fates of these two variants raise a clear question: the 180nt_dup carries a substantially longer insertion, introducing 23–25 extra potential O-linked glycosylation sites in the G protein ectodomain (Saikusa et al., 2014), a feature that might be expected to enhance immune shielding. Yet it is this variant, not the shorter 111nt_dup, that has disappeared from global surveillance. Glycosylation site count alone appears to be a poor predictor of fitness. Several structural constraints may account for this discrepancy. First, the 180nt_dup lineage carries the N90 allele (0% predicted O-glycosylation at position 90), eliminating a glycan anchor immediately upstream of the insertion zone that the 111nt_dup retains; if glycosylation occurs at this site *in vivo*, the resulting gap could disrupt the continuity of the glycan shield across the mucin-like domain. Second, the 180nt_dup adds approximately 60 amino acids to the G protein ectodomain, roughly 23 residues more than the 111nt_dup (~37 amino acids); this additional length may impose greater constraints on protein folding, trafficking, or incorporation into the viral envelope. Third, the higher O-glycan density in the 180nt_dup variant may restrict the exposure of cell-attachment motifs within the mucin-like domain, thereby offsetting the immune-shielding benefit conferred by additional glycosylation. The 111nt_dup may thus strike a more effective balance between antigenic diversification and functional constraint, owing to its moderate insertion length. These structural hypotheses remain to be tested experimentally.

To examine the molecular basis of these divergent evolutionary trajectories, we analyzed the predicted O-glycosylation landscape of the G protein ectodomain across the three A2.2.2 variant groups. A lineage-specific divergence was observed at alignment position 90, immediately upstream of the duplication insertion zone. Non-duplicated strains showed a polymorphic state at this site, with a predicted O-glycosylation frequency of 43.8%. The two duplication lineages were each essentially monomorphic but diverged in opposite directions: the 180nt_dup lineage was fixed for an asparagine residue (N90), with no predicted O-glycosylation at this position (0% frequency); the 111nt_dup lineage, in contrast, showed near-complete fixation of a serine residue (S90), with predicted O-glycosylation at this position in 98.9% of strains. Pairwise comparisons indicated that these amino acid distribution differences were significant across all three groups (all *p* < 0.001). The temporal coupling between the S90 allele and the 111nt_dup variant is consistent with a functional role for this site: S90 frequency remained below 10% before 2015, then rose in parallel with 111nt_dup prevalence, reaching 98.5% in 2025, while the 180nt_dup lineage, fixed for the N90 allele, declined steadily and was no longer detected in global sequence data after 2021.

Extensive O-glycosylation of the hMPV G protein is a recognized mechanism for modulating host antibody access to neutralizing epitopes (Schildgen et al., 2011). These O-glycosylation assignments are computational predictions from NetOGlyc 4.0 and have not been confirmed by mass spectrometry, enzymatic digestion, or lectin-binding assays; as such, they represent hypothesis-generating observations rather than established biological facts. We propose that the predicted S90 glycosylation site, located immediately upstream of the 13 predicted O-glycosylation sites densely clustered within the 111-nucleotide insertion (Piñana et al., 2014), may contribute to the functional properties of the extended mucin-like domain. The 180nt_dup variant, despite carrying a longer insertion with more predicted glycosylation sites (*n* = 24), lacks this upstream modification. The synchronous rise of the S90 allele and the 111nt_dup lineage from 2015 to 2025 is consistent with a hypothesized co-evolution in which the predicted glycosylation site and the duplication together conferred a selective advantage. The intermediate S90 frequency (43.8%) in non-duplicated A2.2.2 strains suggests that serine at this position was already present as a standing polymorphism before the 111nt_dup lineage emerged, and that this pre-existing S90 may have facilitated establishment of the duplication. The temporal order of S90 fixation relative to the 111-nt insertion, however, cannot be definitively resolved given the scarcity of early 111nt_dup sequences from 2013–2015. The epidemiological data—the near-complete fixation of S90 in 111nt_dup, the exclusive N90 status of the declining 180nt_dup lineage, and the tight temporal coupling between allele frequency and variant prevalence—are consistent with a model in which S90-mediated O-glycosylation, if biologically realized, contributed to the selective advantage that drove the 111nt_dup lineage to global predominance, though this remains to be tested experimentally.

The population history reconstructed from the G gene sequences provides a temporal and ecological framework for the molecular findings described above. The estimated evolutionary rate of the 111nt_dup lineage (4.438 × 10^−3^ substitutions/site/year) is consistent with sustained selective pressure on the attachment glycoprotein (De Graaf et al., 2008; Padhi and Verghese, 2008). The tMRCA, dated to approximately mid-2013 (95% HPD: 2012.15–2014.52), places the origin of this lineage roughly 2 years before its first detection in global surveillance data in 2015, a lag consistent with low-level undetected circulation prior to the onset of measurable population expansion (Piñana et al., 2014; Saikusa et al., 2014).

The Bayesian Skyline Plot reveals two epidemiologically significant features. First, a severe population bottleneck coincided with early 2020 through mid-2021. The timing of this contraction corresponds closely with the stringent non-pharmaceutical interventions implemented globally during the COVID-19 pandemic and matches the well-documented suppression of seasonal respiratory virus circulation during this period (Foley et al., 2022; Ye et al., 2024). Reduced global sequencing submissions during the acute phases of the pandemic may have amplified the apparent severity of this bottleneck in the Skyline reconstruction; however, the well-documented suppression of hMPV and other seasonal respiratory viruses during this period indicates that the contraction reflects a genuine biological decline, albeit one whose visual magnitude in the phylodynamic reconstruction may be compounded by concurrent sampling gaps. Second, a rapid expansion followed from late 2021 through early 2022 as these interventions were relaxed (Foley et al., 2023). A transient decline in population-level immunity, the “immunity debt” described in recent respiratory virus literature, offers a plausible ecological explanation, though its role in hMPV resurgence has not been directly quantified (Cohen et al., 2021; Cohen et al., 2023). The return of the 111nt_dup lineage to a stable population size comparable to its pre-pandemic level by late 2022 suggests that the competitive advantage of the S90-mediated glycosylation profile and the 111-nucleotide insertion was not eroded by the bottleneck. The lineage retained its epidemiological fitness through the population crash and re-expanded to reoccupy its prior ecological niche.

Several limitations should be noted. First, the O-glycosylation profiles reported here are computational predictions (NetOGlyc 4.0) and require validation by mass spectrometry, enzymatic digestion with O-glycosidase, or lectin-binding assays before the biological role of the S90 site can be considered established. Furthermore, in silico glycosylation predictions do not account for the influence of local protein folding, tertiary conformation of the folded virion ectodomain, or host cell-specific glycosyltransferase expression on the actual bioavailability of predicted modification sites. As such, the O-glycosylation patterns presented here should be interpreted as hypothesis-generating computational observations, and the proposed mechanistic link between S90-mediated glycosylation and 111nt_dup fitness remains to be tested experimentally. Second, the local genomic dataset is modest in size, which may limit the detection of low-frequency transitional variants and precludes robust statistical comparison of genotype-specific clinical severity. Third, this modest sample size also leaves unresolved whether the observed genotype turnover reflects differential fitness advantages of genotype B2 or stochastic bottlenecking of low-frequency genotype A lineages during inter-seasonal latency. Despite these limitations, our findings indicate that the rise of the A2.2.2 111nt_dup lineage to dominance is better explained by lineage-specific molecular adaptation than by neutral drift. The evolutionary trajectory of this variant highlights the importance of sustained genomic surveillance, since modifications in the attachment glycoprotein capable of altering immune recognition are directly relevant to hMPV vaccine design and monoclonal antibody development. Given the rapid lineage replacement observed in the G protein mucin-like domain, we recommend that future vaccine strain surveillance track G gene duplication status and the S90/N90 polymorphism as molecular early-warning markers for emerging lineages.

## Data Availability

The datasets presented in this study can be found in online repositories. The names of the repository/repositories and accession number(s) can be found in the article/Supplementary material.

## Footnotes

1
https://chiplot.online/tvbot.html

2
https://services.healthtech.dtu.dk/services/NetOGlyc-4.0/
