# Supplementary material for: Genotype turnover and global evolutionary dynamics of G gene duplication variants in human metapneumovirus: insights from prospective surveillance in Nantong, China, 2024–2025
Source: Front Microbiol. 2026 Aug 12;17:1915595. doi: 10.3389/fmicb.2026.1915595 (PMC13506792; doi:10.3389/fmicb.2026.1915595)
Supplement: Supplementary file 1 [file Data_Sheet_2.pdf]

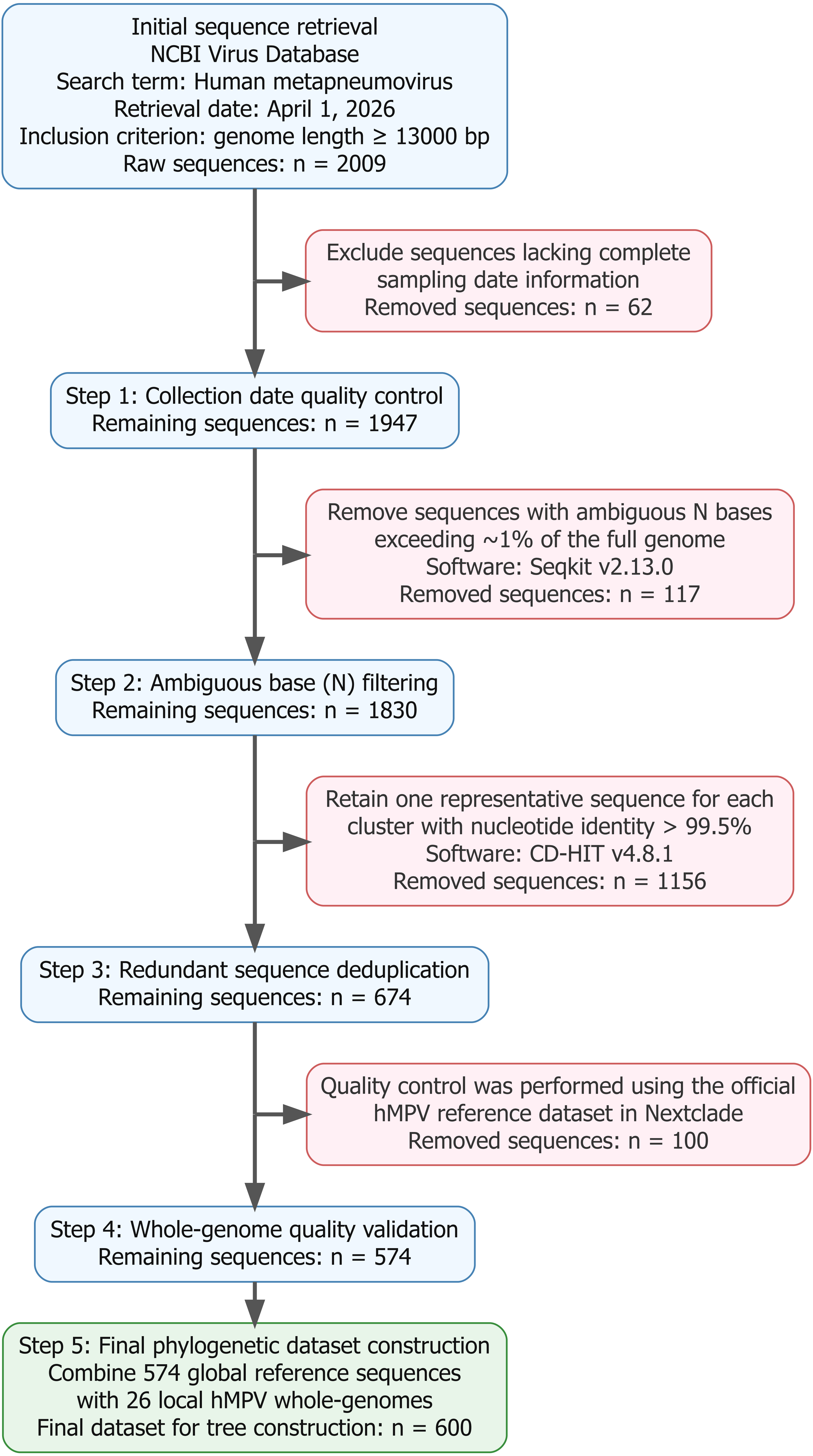
Initial sequence retrieval
NCBI Virus Database
Search term: Human metapneumovirus
Retrieval date: April 1, 2026
Inclusion criterion: genome length ≥ 13000 bp
Raw sequences: n = 2009
Exclude sequences lacking complete sampling date information
Removed sequences: n = 62
Step 1: Collection date quality control
Remaining sequences: n = 1947
Remove sequences with ambiguous N bases exceeding ~1% of the full genome
Software: Seqkit v2.13.0
Removed sequences: n = 117
Step 2: Ambiguous base (N) filtering
Remaining sequences: n = 1830
Retain one representative sequence for each cluster with nucleotide identity > 99.5%
Software: CD-HIT v4.8.1
Removed sequences: n = 1156
Step 3: Redundant sequence deduplication
Remaining sequences: n = 674
Quality control was performed using the official hMPV reference dataset in Nextclade
Removed sequences: n = 100
Step 4: Whole-genome quality validation
Remaining sequences: n = 574
Step 5: Final phylogenetic dataset construction
Combine 574 global reference sequences with 26 local hMPV whole-genomes
Final dataset for tree construction: n = 600
