# Supplementary material for: Genotype turnover and global evolutionary dynamics of G gene duplication variants in human metapneumovirus: insights from prospective surveillance in Nantong, China, 2024–2025
Source: Front Microbiol. 2026 Aug 12;17:1915595. doi: 10.3389/fmicb.2026.1915595 (PMC13506792; doi:10.3389/fmicb.2026.1915595)
Supplement: Supplementary file 2 [file Data_Sheet_3.pdf]

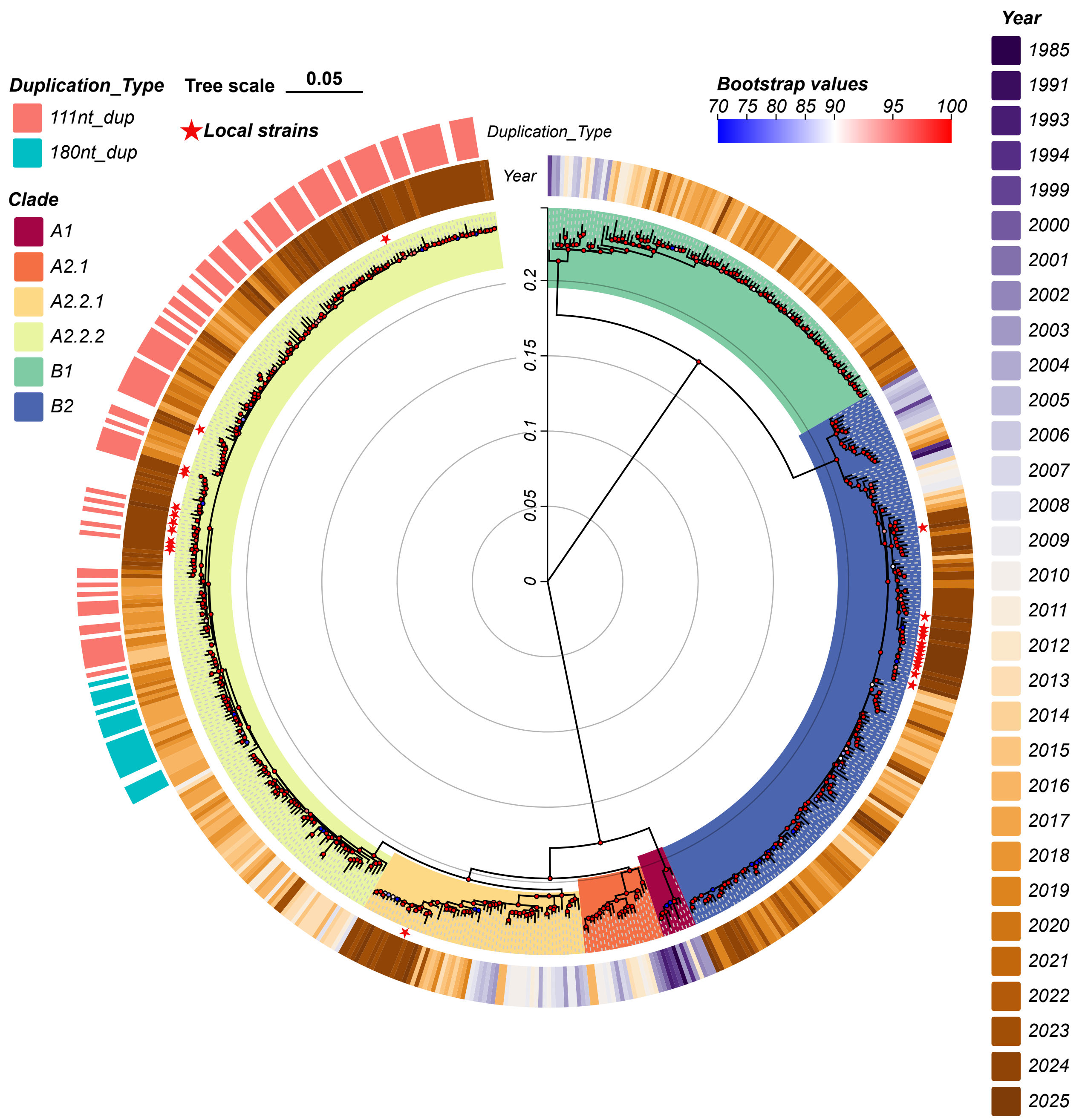
Duplication_Type
111nt_dup
180nt_dup
Tree scale
0.05
Local strains
Clade
A1
A2.1
A2.2.1
A2.2.2
B1
B2
Duplication_Type
Year
0
0.05
0.1
0.15
0.2
Bootstrap values
70
75
80
85
90
95
100
Year
1985
1991
1993
1994
1999
2000
2001
2002
2003
2004
2005
2006
2007
2008
2009
2010
2011
2012
2013
2014
2015
2016
2017
2018
2019
2020
2021
2022
2023
2024
2025
