# Supplementary material for: Genotype turnover and global evolutionary dynamics of G gene duplication variants in human metapneumovirus: insights from prospective surveillance in Nantong, China, 2024–2025
Source: Front Microbiol. 2026 Aug 12;17:1915595. doi: 10.3389/fmicb.2026.1915595 (PMC13506792; doi:10.3389/fmicb.2026.1915595)
Supplement: Supplementary file 3 [file Data_Sheet_4.pdf]

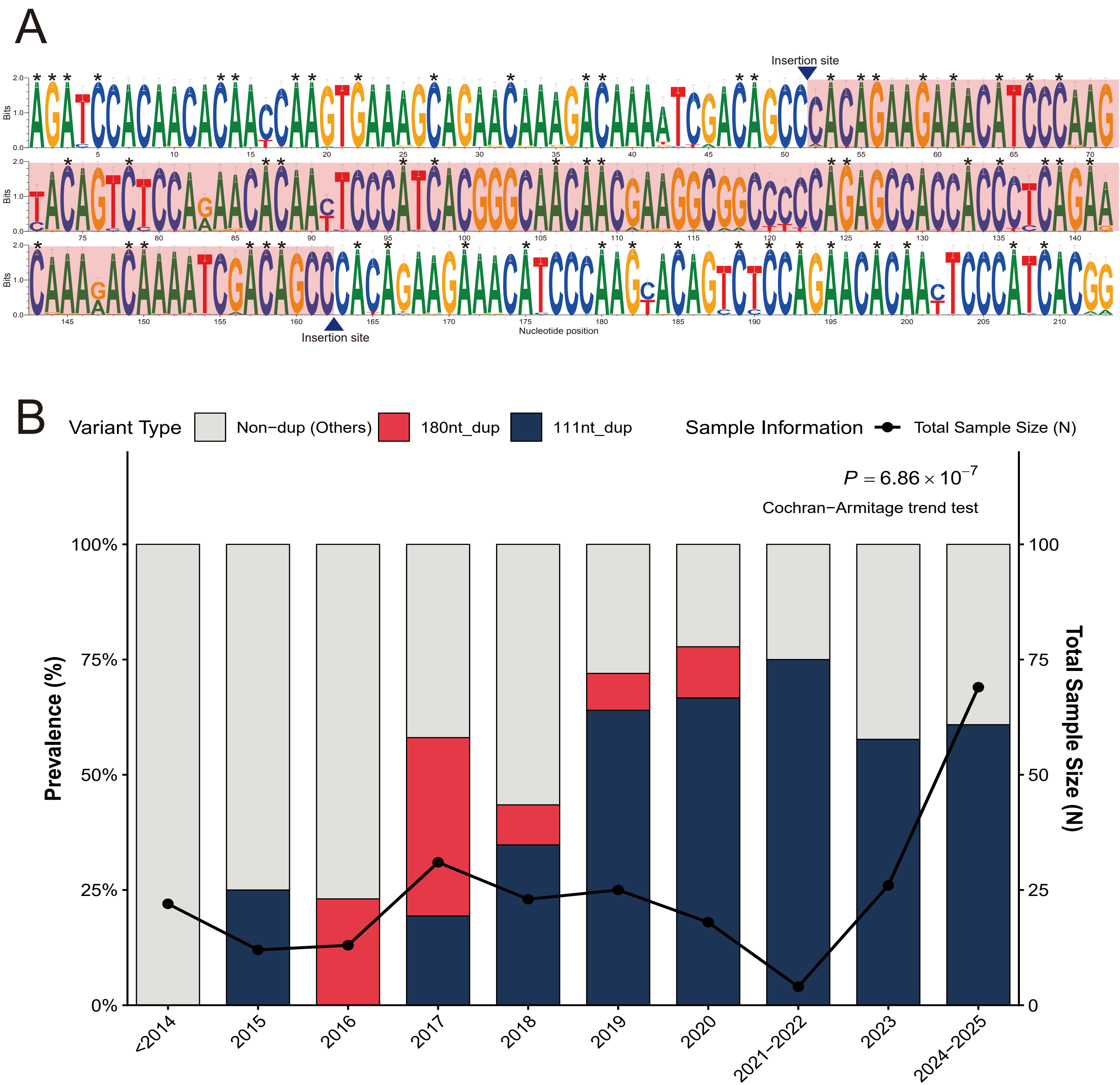
A
Insertion site
Bits
Nucleotide position
Insertion site
B
Variant Type
Non−dup (Others)
180nt_dup
111nt_dup
Sample Information
Total Sample Size (N)
$P = 6.86 \times 10^{-7}$
Cochran−Armitage trend test
Prevalence (%)
100%
75%
50%
25%
0%
Total Sample Size (N)
100
75
50
25
0
<2014
2015
2016
2017
2018
2019
2020
2021−2022
2023
2024−2025
