# Supplementary material for: Genotype turnover and global evolutionary dynamics of G gene duplication variants in human metapneumovirus: insights from prospective surveillance in Nantong, China, 2024–2025
Source: Front Microbiol. 2026 Aug 12;17:1915595. doi: 10.3389/fmicb.2026.1915595 (PMC13506792; doi:10.3389/fmicb.2026.1915595)
Supplement: Supplementary file 4 [file Data_Sheet_5.pdf]

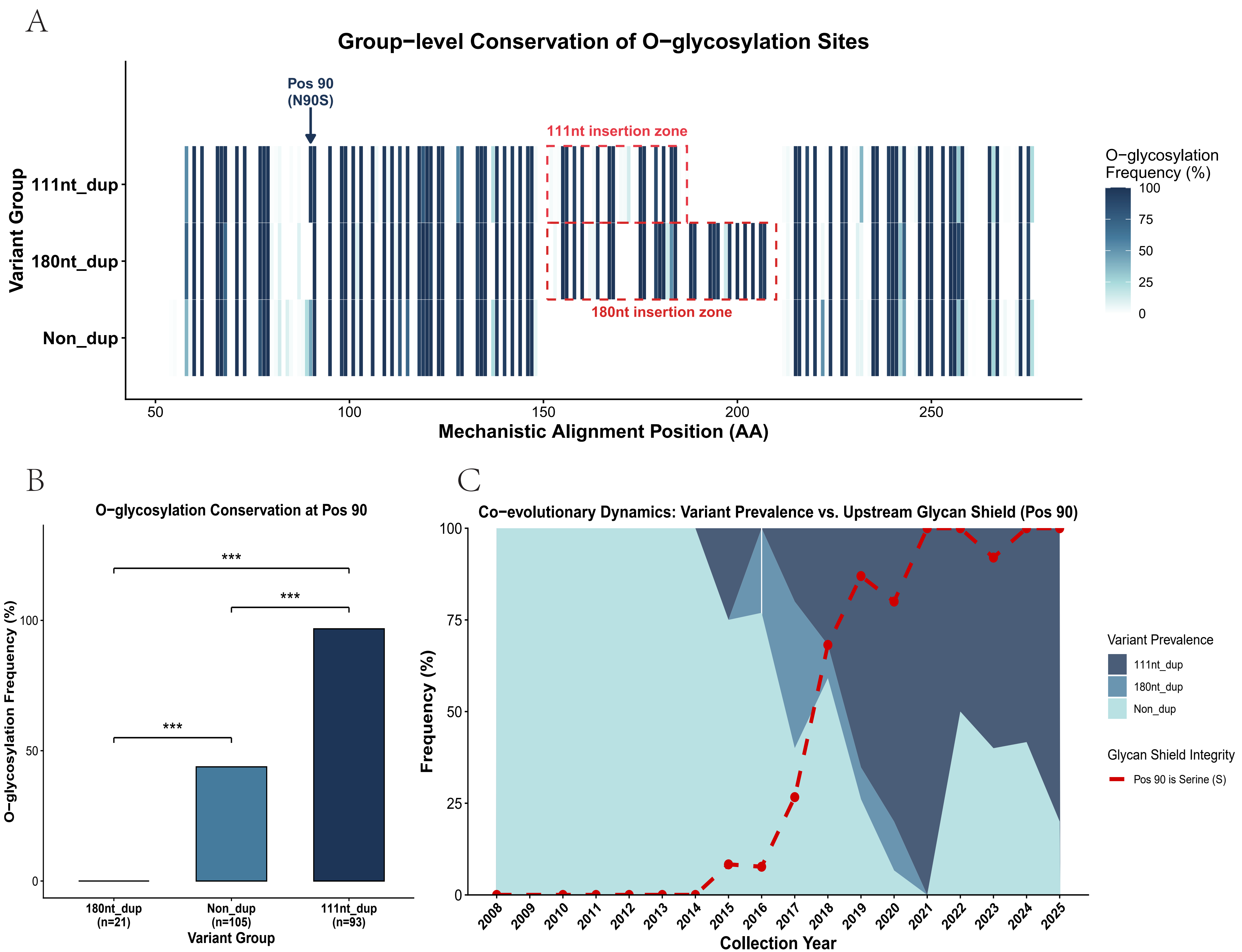
A
Group−level Conservation of O−glycosylation Sites
Pos 90 (N90S)
111nt insertion zone
180nt insertion zone
Variant Group
111nt_dup
180nt_dup
Non_dup
Mechanistic Alignment Position (AA)
50
100
150
200
250
O−glycosylation Frequency (%)
100
75
50
25
0
B
O−glycosylation Conservation at Pos 90
O−glycosylation Frequency (%)
***
***
***
180nt_dup (n=21)
Non_dup (n=105)
111nt_dup (n=93)
Variant Group
C
Co−evolutionary Dynamics: Variant Prevalence vs. Upstream Glycan Shield (Pos 90)
Frequency (%)
Collection Year
2008
2009
2010
2011
2012
2013
2014
2015
2016
2017
2018
2019
2020
2021
2022
2023
2024
2025
Variant Prevalence
111nt_dup
180nt_dup
Non_dup
Glycan Shield Integrity
Pos 90 is Serine (S)
