# Supplementary material for: Genotype turnover and global evolutionary dynamics of G gene duplication variants in human metapneumovirus: insights from prospective surveillance in Nantong, China, 2024–2025
Source: Front Microbiol. 2026 Aug 12;17:1915595. doi: 10.3389/fmicb.2026.1915595 (PMC13506792; doi:10.3389/fmicb.2026.1915595)
Supplement: Supplementary file 5 [file Data_Sheet_6.pdf]

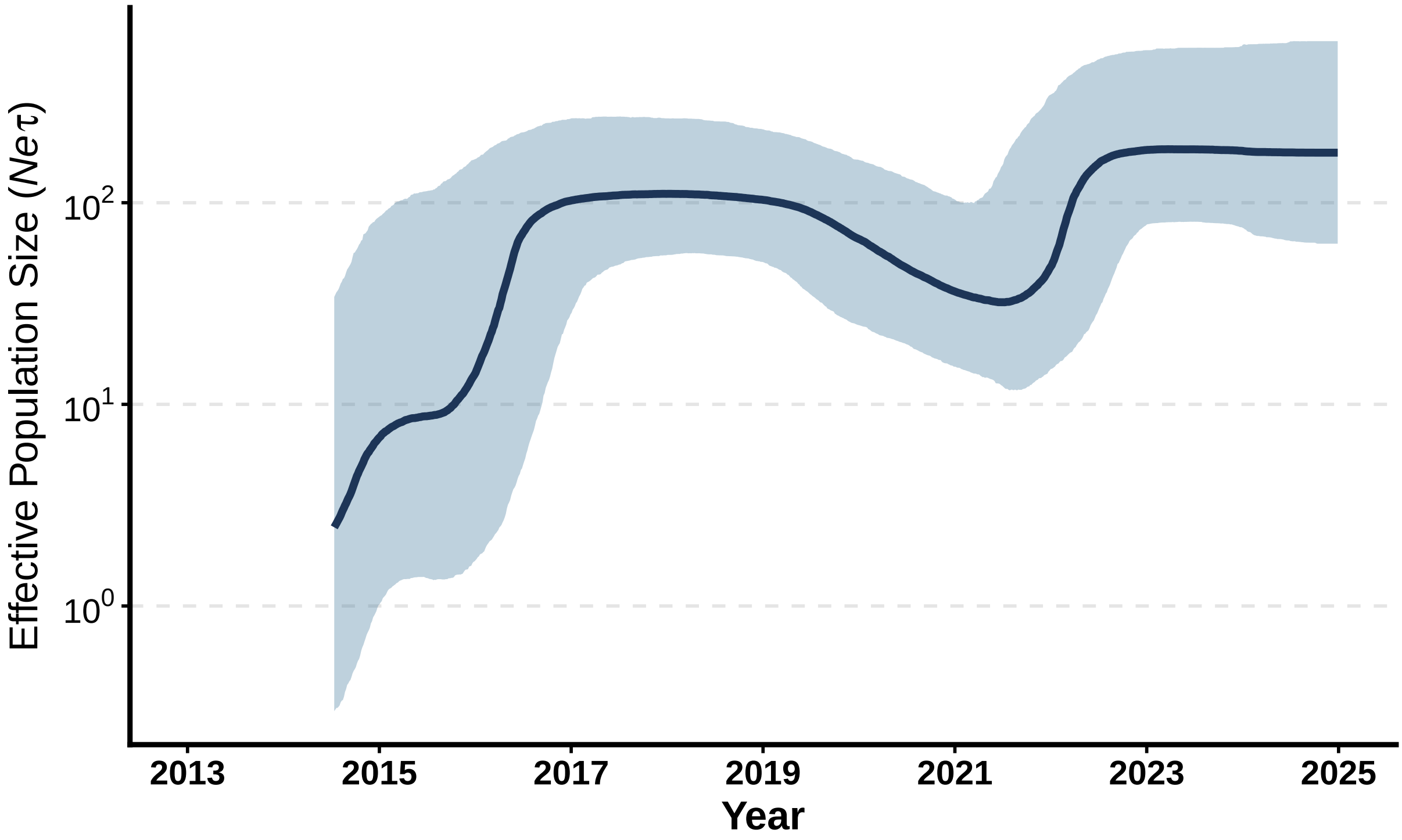
Effective Population Size ($Ne\tau$)
10^2
10^1
10^0
2013
2015
2017
2019
2021
2023
2025
Year
