# Supplementary material for: Genotype turnover and global evolutionary dynamics of G gene duplication variants in human metapneumovirus: insights from prospective surveillance in Nantong, China, 2024–2025
Source: Front Microbiol. 2026 Aug 12;17:1915595. doi: 10.3389/fmicb.2026.1915595 (PMC13506792; doi:10.3389/fmicb.2026.1915595)
Supplement: Supplementary file 6 [file Image_1.pdf]

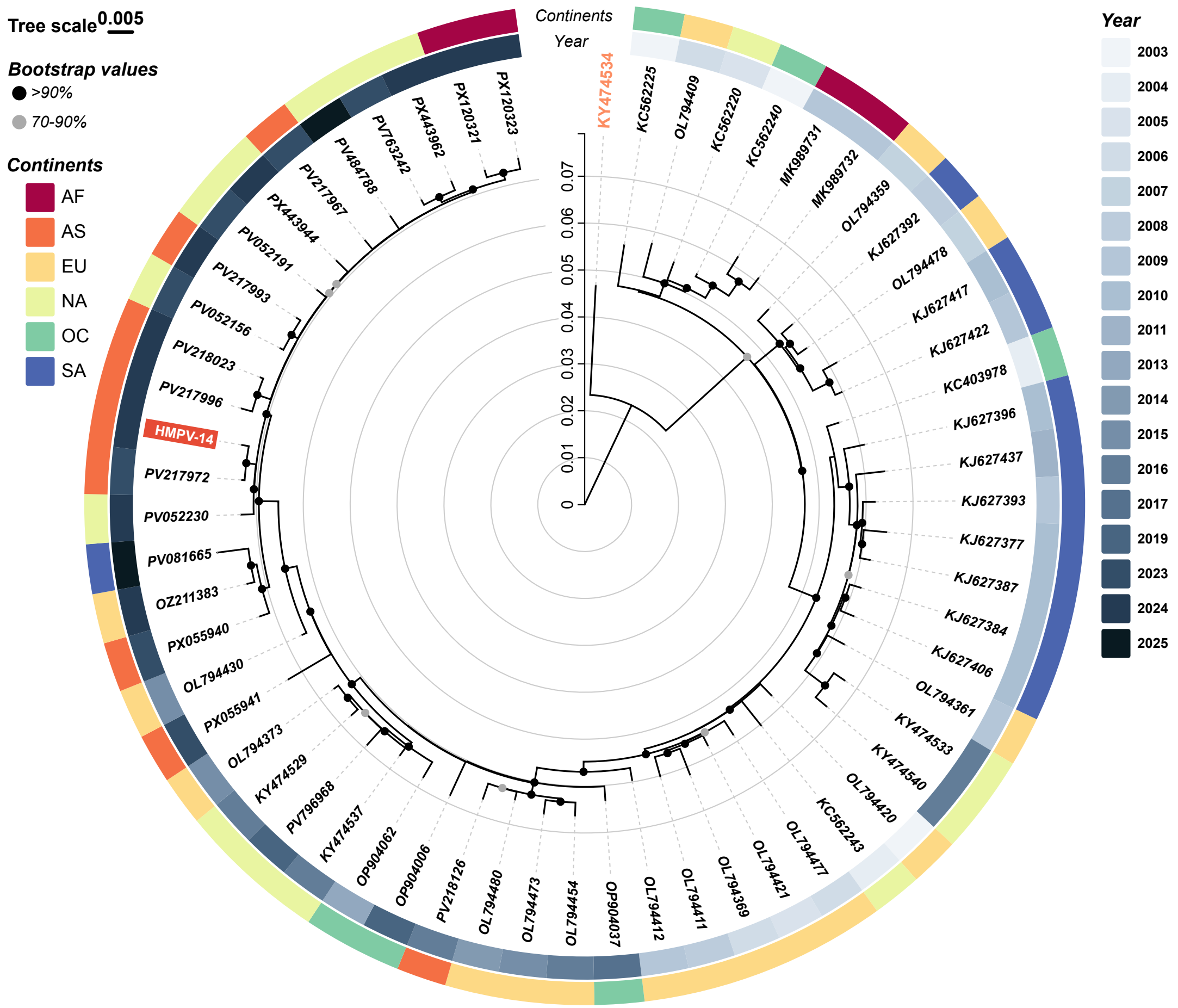

Supplementary Figure S1. Maximum-likelihood phylogenetic tree of full-length hMPV A2.2.1 genomes. The tree was reconstructed using IQ-TREE 2, with the optimal nucleotide substitution model selected by ModelFinder according to the Bayesian Information Criterion. Branch support was assessed using 1,000 ultrafast bootstrap replicates. The Nantong sequence generated in this study, HMPV-14, is highlighted in red. The inner and outer annotation rings indicate the year of sample collection and continent of origin, respectively. Black and gray circles indicate bootstrap support values of >90% and 70–90%, respectively. The orange tip label denotes the outgroup sequence used to root the tree. The scale bar represents nucleotide substitutions per site. AF, Africa; AS, Asia; EU, Europe; NA, North America; OC, Oceania; SA, South America.

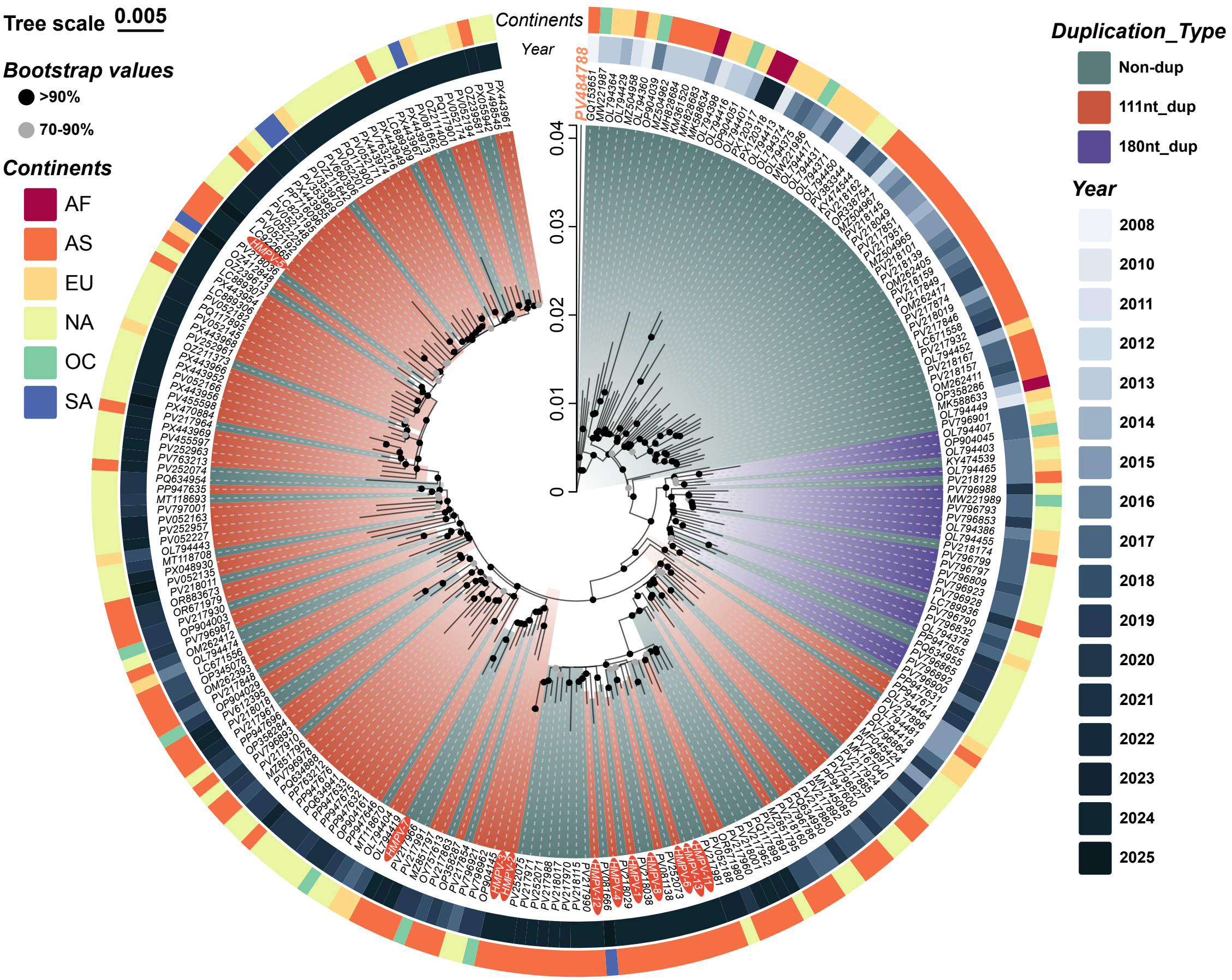

Supplementary Figure S2. Maximum-likelihood phylogenetic tree of full-length hMPV A2.2.2 genomes annotated by G gene duplication type. The tree was reconstructed using IQ-TREE 2, with the optimal nucleotide substitution model selected by ModelFinder according to the Bayesian Information Criterion. Branch support was assessed using 1,000 ultrafast bootstrap replicates. The 11 Nantong sequences generated in this study are highlighted in red. The colored inner sectors indicate the G gene duplication type: non-dup, 111nt_dup, or 180nt_dup. The two outer annotation rings indicate the year of sample collection and continent of origin, respectively. Black and gray circles indicate bootstrap support values of >90% and 70–90%, respectively. The orange tip label denotes the outgroup sequence used to root the tree. The scale bar represents nucleotide substitutions per site. AF, Africa; AS, Asia; EU, Europe; NA, North America; OC, Oceania; SA, South America..

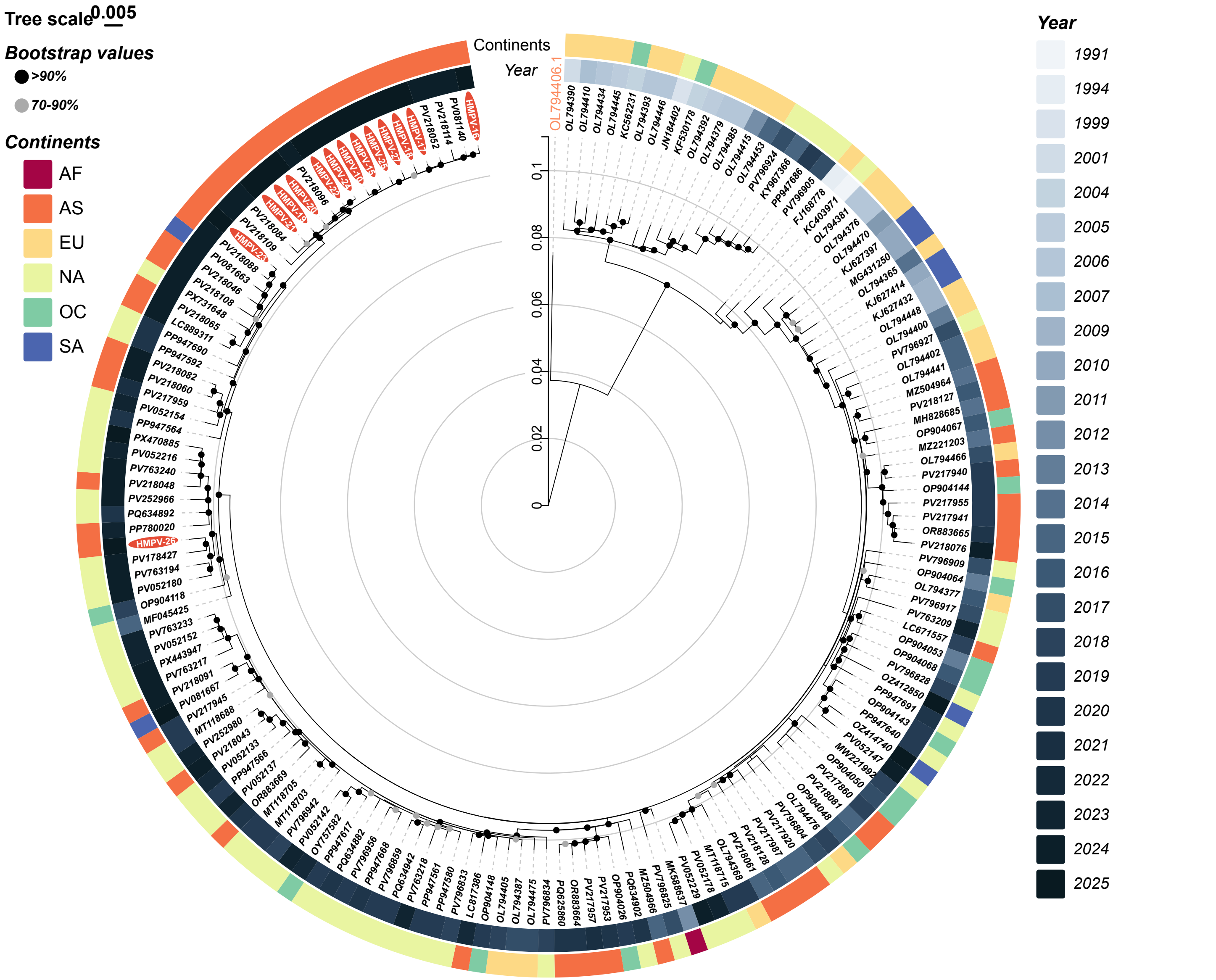

Supplementary Figure S3. Maximum-likelihood phylogenetic tree of full-length hMPV B2 genomes. The tree was reconstructed using IQ-TREE 2, with the optimal nucleotide substitution model selected by ModelFinder according to the Bayesian Information Criterion. Branch support was assessed using 1,000 ultrafast bootstrap replicates. The 14 Nantong sequences generated in this study are highlighted in red. The inner and outer annotation rings indicate the year of sample collection and continent of origin, respectively. Black and gray circles indicate bootstrap support values of >90% and 70–90%, respectively. The orange tip label denotes the outgroup sequence used to root the tree. The scale bar represents nucleotide substitutions per site. AF, Africa; AS, Asia; EU, Europe; NA, North America; OC, Oceania; SA, South America.
