# Supplementary material for: Genotype turnover and global evolutionary dynamics of G gene duplication variants in human metapneumovirus: insights from prospective surveillance in Nantong, China, 2024–2025
Source: Front Microbiol. 2026 Aug 12;17:1915595. doi: 10.3389/fmicb.2026.1915595 (PMC13506792; doi:10.3389/fmicb.2026.1915595)
Supplement: Supplementary file 7 [file Data_Sheet_1.zip › S1-S3/S1_A2.2.1.pdf]

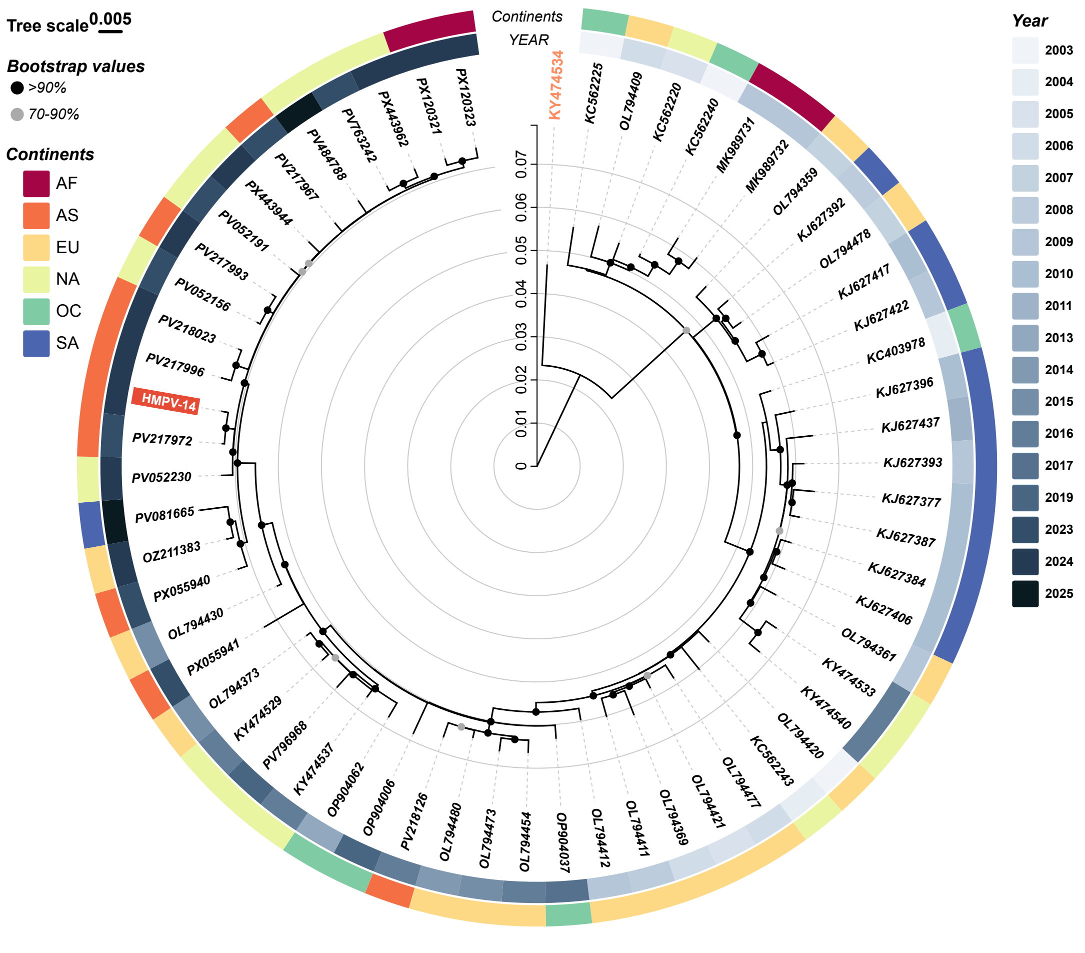

Tree scale 0.005
Bootstrap values
>90%
70-90%
Continents
AF
AS
EU
NA
OC
SA
Year
2003
2004
2005
2006
2007
2008
2009
2010
2011
2013
2014
2015
2016
2017
2019
2023
2024
2025
Continents
YEAR
0
0.01
0.02
0.03
0.04
0.05
0.06
0.07
KY474534
KC562225
OL794409
KC562220
KC562240
MK989731
MK989732
OL794359
KJ627392
OL794478
KJ627417
KJ627422
KC403978
KJ627396
KJ627437
KJ627393
KJ627377
KJ627387
KJ627384
KJ627406
OL794361
KY474533
KY474540
OL794420
KC562243
OL794477
OL794421
OL794369
OL794411
OL794412
OP904037
OL794454
OL794473
OL794480
PV218126
OP904006
OP904062
KY474537
PV796968
KY474529
OL794373
PX055941
OL794430
PX055940
OZ211383
PV081665
PV052230
PV217972
HMPV-14
PV217996
PV218023
PV052156
PV217993
PV052191
PX443944
PV217967
PV484788
PV763242
PX443962
PX120321
PX120323
