# Supplementary material for: Genotype turnover and global evolutionary dynamics of G gene duplication variants in human metapneumovirus: insights from prospective surveillance in Nantong, China, 2024–2025
Source: Front Microbiol. 2026 Aug 12;17:1915595. doi: 10.3389/fmicb.2026.1915595 (PMC13506792; doi:10.3389/fmicb.2026.1915595)
Supplement: Supplementary file 7 [file Data_Sheet_1.zip › S1-S3/S3_B2.pdf]

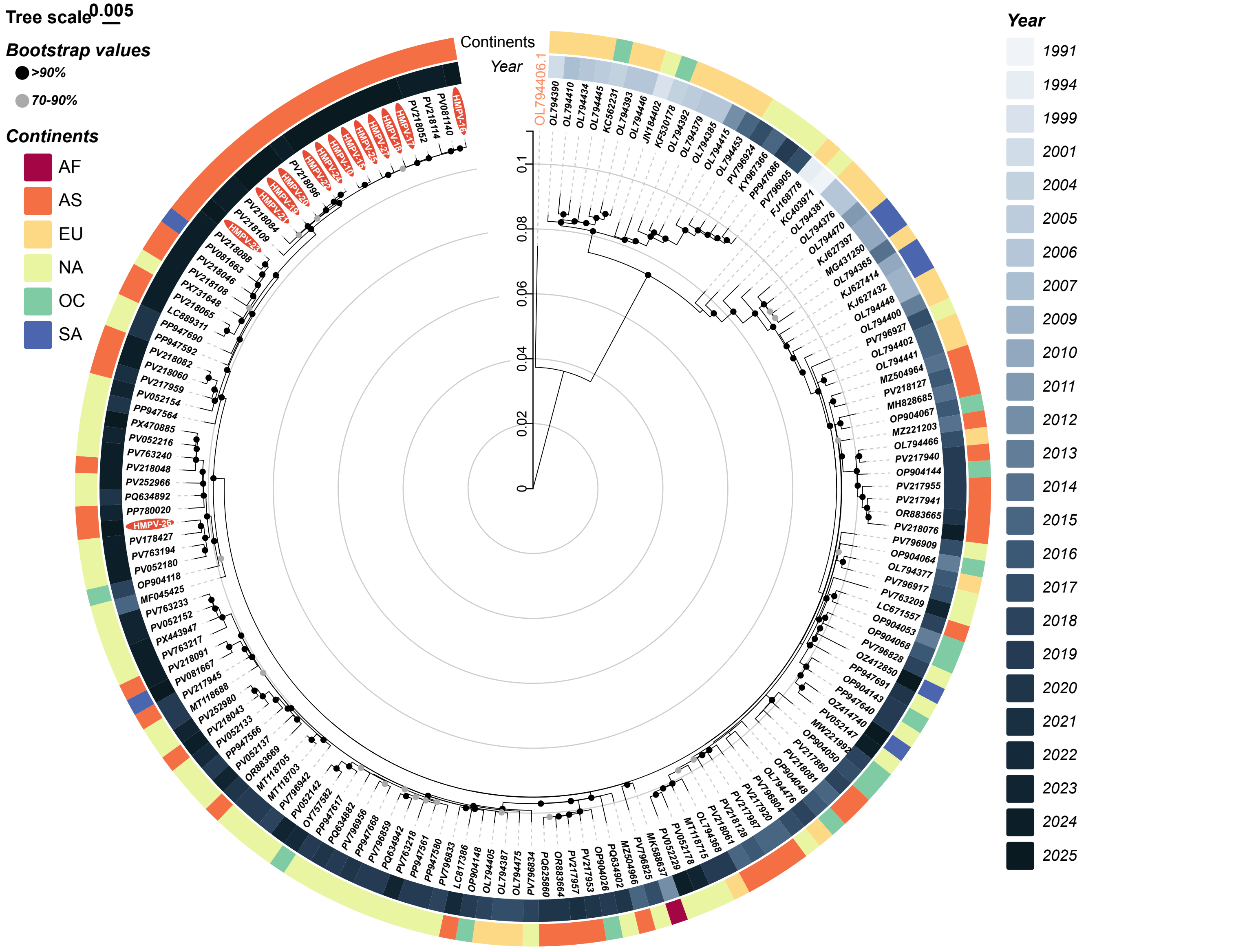
Tree scale 0.005
Bootstrap values
>90%
70-90%
Continents
AF
AS
EU
NA
OC
SA
Continents
Year
OL794406.1
0
0.02
0.04
0.06
0.08
0.1
Year
1991
1994
1999
2001
2004
2005
2006
2007
2009
2010
2011
2012
2013
2014
2015
2016
2017
2018
2019
2020
2021
2022
2023
2024
2025
